# Novel Variant of New Delhi Metallo-β-lactamase, NDM-20, in *Escherichia coli*

**DOI:** 10.3389/fmicb.2018.00248

**Published:** 2018-02-21

**Authors:** Zhihai Liu, Jiyun Li, Xiaoming Wang, Dejun Liu, Yuebin Ke, Yang Wang, Jianzhong Shen

**Affiliations:** ^1^Beijing Key Laboratory of Detection Technology for Animal-Derived Food Safety, College of Veterinary Medicine, China Agricultural University, Beijing, China; ^2^Key Laboratory of Genetics & Molecular Medicine of Shenzhen, Shenzhen Center for Disease Control and Prevention, Shenzhen, China

**Keywords:** NDM-20, carbapenemase, ST1114, IncX3, kinetic parameters

## Abstract

The spread of carbapenem-resistant Enterobacteriaceae (CRE) mediated by New Delhi metallo-β-lactamase (NDM) poses a serious challenge to clinicians and has become a major public health concern. NDM has been evolving into variants that possess different hydrolysis activity toward antibiotics, so as to affect treatment strategy. In addition, very few studies on NDM variants have focused on animal-derived bacterial isolates. Our study reports a novel NDM variant, NDM-20, in an isolate of *Escherichia coli* CCD1 recovered from the food animal swine in China. The isolate that was assigned to ST1114, exhibited high level resistance to all β-lactams tested, including aztreonam and carbapenems. The gene of *bla*_NDM-20_ was located on an IncX3-type plasmid, surrounded by multiple insertion sequences. Sequencing analysis demonstrated that *bla*_NDM-20_ contained three point mutations at positions 262 (G→T), 460 (A→C), and 809 (G→A), compared with *bla*_NDM-1_, and just one point mutation at position 809 (G→A), relative to *bla*_NDM-5_. Functional analysis revealed that the *bla*_NDM-20_ transformant, DH5α+pHSG398/NDM-20, exhibited a higher resistance to ertapenem than that of *bla*_NDM-1_ transformant DH5α+pHSG398/NDM-1. Kinetic parameter analysis showed that NDM-20 had increased enzymatic activity against some penicillins and cephalosporins but decreased carbapenemase activity relative to NDM-5. The identification of NDM-20 further confirms the evolution and prevalence of NDM variants in bacteria of food-animal origin.

## Introduction

The global dissemination of carbapenem-resistant Enterobacteriaceae (CRE) has become a major threat to public health. With the increasing global prevalence of New Delhi metallo-β-lactamase (NDM)-producing bacteria, which are highly disseminated in China (Khan et al., 2017), available antibiotics are becoming less effective in the treatment of drug-resistant bacterial infections. Since NDM was first reported in India (Yong et al., 2009), the sequences of 18 NDM variants (NDM-19 unpublished) have been reported. NDMs may be evolving diverse hydrolytic activity toward β-lactams, resulting from amino acid substitutions or insertions (Khan et al., 2017), subsequently, it may affect treatment strategy. In addition, these bacteria producing NDM variants are disseminated not only among humans, but also in food animals (Wang et al., 2017). Even though food-producing animals serve as the major reservoir of NDM-producing bacteria (Madec et al., 2017), apart from our previous description of NDM-17 (Liu et al., 2017), studies on NDM variants of animal-derived bacterial isolates are limited. So, it is important and necessary to monitor NDM evolution in food animals. Here, a novel NDM variant, NDM-20, was identified in an *Escherichia coli* strain isolated from swine, and was subjected to in-depth molecular characterization.

## Materials and Methods

### Isolation and Identification of the Bacterial Strain and Phenotypic Screening for MBL Production

To better understand the prevalence of CRE, a routine surveillance of antimicrobial resistance in bacteria of food-producing animal origin was carried out in Shandong province, China, in 2016. The swine anal swab samples were collected and enriched in 5 mL of brain heart infusion broth before being plated onto CHROMagar KPC medium (CHROMagar, Paris, France) for CRE selection. Then, the resistant strains were identified on MHA medium containing 1 mg/L of meropenem. Strain CCD1 was isolated from a swine fecal swab sample from a commercial farm. Identification of the bacterial species was performed by MALDI-TOF MS (Bruker Daltonik, Bremen, Germany) and 16S rRNA sequencing, as described previously (Wang et al., 2013). To detect carbapenemase production, the modified Hodge test was carried out using imipenem and meropenem disks, and for MBL production test, the combined-disk was performed by imipenem with and without EDTA.

### Antimicrobial Susceptibility Testing and Detection of Antibiotic Resistance Genes

Antimicrobial susceptibility testing of isolates was carried out using the broth microdilution method, and the results in triplicate with best repeatability were interpreted according to the CLSI and EUCAST (version 7.1) guidelines (Clinical and Laboratory Standards Institute [CLSI], 2015; EUCAST, 2017). The tested isolates and antimicrobial agents are listed in **Table 1**, and *E. coli* ATCC 25922 was used as a quality control strain. Carbapenemase-encoding genes *bla*_KPC_, *bla*_V IM_, *bla*_IMP_, *bla*_NDM_, *bla*_OXA-23-like_, *bla*_OXA-58-like_, *bla*_OXA-48-like_, *bla*_IND_, *bla*_KHM_, *bla*_DIM_, *bla*_GIM_, *bla*_SIM_, and *bla*_SPM_, and β-lactamase-encoding genes *bla*_TEM_, *bla*_SHV_, and *bla*_CTX-M_ were screened as previously described (Dallenne et al., 2010; Poirel et al., 2011; Zhang et al., 2017b), and *bla*_NDM_ was screened by primer preA and preB in **Supplementary Table S1**. PCR amplicons were sequenced and then analyzed against the GenBank database.

**Table 1:** The resistance gene(s) in CCD1 and β-lactam MICs for the New Delhi metallo-β-lactamase (NDM)-carrying *Escherichia coli* clinical isolate, transconjugants, and transformants.

Antibiotics*^a^*	MIC (mg/L) for strains						
	CCD1	20J3	J53	DH5α+ pHSG398	DH5α+ pHSG398/NDM-20	DH5α+ pHSG398/NDM-5	DH5α+ pHSG398/NDM-1
Ampicillin	>512	>512	2	2	512	512	512
Amoxicillin	>512	512	16	1	512	512	512
Amoxicillin+CLA	128	128	16	2	128	128	128
Aztreonam	256	0.12	0.06	0.06	0.03	0.03	0.06
Ceftazidime	>512	>512	1	0.5	512	256	128
Cefoxitin	512	512	8	4	256	128	256
Cefazolin	>512	>512	4	2	256	256	256
Cefotaxime	512	512	0.12	0.03	32	32	32
Cefepime	512	256	0.12	0.015	4	2	2
Ertapenem	128	64	0.06	0.015	1	2	0.25
Imipenem	128	64	0.5	0.125	4	4	4
Meropenem	128	64	0.12	0.03	2	4	2
Penicillin G	>512	>512	32	16	512	512	512
Piperacillin	>512	>512	16	0.5	16	16	16
Piperacillin+TZB	>512	>512	16	0.5	16	16	16
Colistin	0.25	0.12	0.25	0.12	0.12	0.12	0.12
Gentamicin	1	2	2	1	1	1	1
Tigecycline	0.06	0.06	0.06	0.06	0.06	0.03	0.03

^a^*CLA, clavulanic acid (fixed concentration of 2 mg/L); TZB, tazobactam (fixed concentration of 4 mg/L)*.

### Conjugation Assay and Location Analysis of *bla*_NDM_

To determine the transferability of *bla*_NDM_, transconjugation was attempted using *E. coli* J53 as the recipient (Liu et al., 2017). Transconjugants were selected on MacConkey agar supplemented with 100 mg/L sodium azide and 1 mg/L meropenem, and confirmed by PCR and sequencing. The transfer frequency was determined from the ratio of transconjugants: donors. Location analysis of *bla*_NDM_ was performed by S1-PFGE and Southern blotting, as described previously (Borgia et al., 2012).

### Resistome and Plasmid Analysis

To further confirm the resistance gene profile of strain CCD1 and to determine the genetic background of *bla*_NDM_, the genomic DNA of strain CCD1, including plasmid pNDM-20, was extracted and subjected to WGS, as reported previously (Liu et al., 2017; Wang et al., 2017). A 250-bp paired-end library was constructed using a NEXT Ultra DNA Library Prep kit (New England Biolabs, E7645L) and an Illumina Hiseq 2500 system (Bionova Biotech, Co., Beijing, China). The raw sequencing data were then *de novo* assembled and examined for the presence of resistance genes using CLC Genomics Workbench 9.0 (CLC Bio, Aarhus, Denmark) against resistance gene database based on CARD. SRST2 was used for MLST analysis of strain CCD1 using the sequence reads (Inouye et al., 2014).

### Cloning of *bla*_NDM_ Genes and Purification of the NDM Proteins

To compare the resistance abilities of *bla*_NDM-20_, *bla*_NDM-5_ and *bla*_NDM-1_, the entire *bla*_NDM-20_, *bla*_NDM-5_, and *bla*_NDM-1_ ORFs were cloned into pHSG398, resulting in recombinant plasmids pHSG398/NDM-20, pHSG398/NDM-5, and pHSG398/NDM-1, respectively (Liu et al., 2017). The plasmids were then transformed into *E. coli* DH5α. To assay the effect on enzymatic activity caused by Arg270His, *bla*_NDM-20_ and *bla*_NDM-5_ minus their signal peptide regions were also cloned into the pET28a expression vector, and the resulting plasmids were transformed into *E. coli* BL21 (DE3) (TransGen Biotech, Beijing, China) (Liu et al., 2017). The corresponding primers were described in **Supplementary Table S1**. All the transformants were confirmed by PCR and sequencing analysis, and their sensitivity to β-lactams were determined as described above. Purification and identification of the NDM proteins was carried out as per the manufacturer’s instructions in the following order: (1) purification of recombinant NDM proteins using Ni-nitrilotriacetic acid agarose; (2) cleavage of His-tags using Turbo TEV protease (Accelagen, San Diego, CA, United States); (3) collection of untagged proteins by an additional passage over Ni-nitrilotriacetic acid agarose; (4) SDS-PAGE analysis to assess the purity of the protein products; (5) measurement of protein concentrations by using a Pierce bicinchoninic acid protein assay kit (Thermo Scientific, Waltham, MA, United States); and (6) monitoring β-lactamase activity using nitrocefin (Oxoid, Ltd., Basingstoke, United Kingdom) (Liu et al., 2017).

### Enzyme Kinetics Assay

To assay the catalytic properties of NDM-20 and NDM-5, a kinetic study was conducted. A SpectraMax M5 multi-detection microplate reader (Molecular Devices, Sunnyvale, CA, United States) was used to measure initial hydrolysis rates in 30 mM phosphate buffer (30 μM Zn^2+^, pH 7.0) at 25°C. The hydrolysis rates were then used to determine the *K_m_* and *k*_cat_ values, as well as the *k*_cat_/*K_m_* ratio, using a Lineweaver–Burk plot. All data used to determine the hydrolysis rates were collected from three individual experiments, and wavelengths and extinction coefficients were applied as previously described (Crowder et al., 1998; Queenan et al., 2010).

### Accession Numbers

The sequence of *bla*_NDM-20_ gene has been deposited in GenBank under accession no. KY654092 (sequence of *bla*_NDM-20_ in **Supplementary data 1**). The complete nucleotide sequence of plasmid pNDM-20 (sequence of pNDM-20 plasmid in **Supplementary data 2**) has been deposited as GenBank accession no. MF458176.

## Results and Discussion

### Strain Features

*Escherichia coli* strain CCD1 was isolated from a swine fecal swab collected from a commercial pig farm in Shandong province, China. The susceptibility testing results showed that *E. coli* CCD1 exhibited high level resistance to all β-lactams examined (**Table 1**), including aztreonam, carbapenems and combinations of penicillins and β-lactamase inhibitors, but susceptible to gentamicin and colistin. The modified Hodge test was positive for carbapenemase, and subsequent positive result from the MBL combined-disk test further suggested MBL production in *E. coli* CCD1.

**FIGURE 1:**
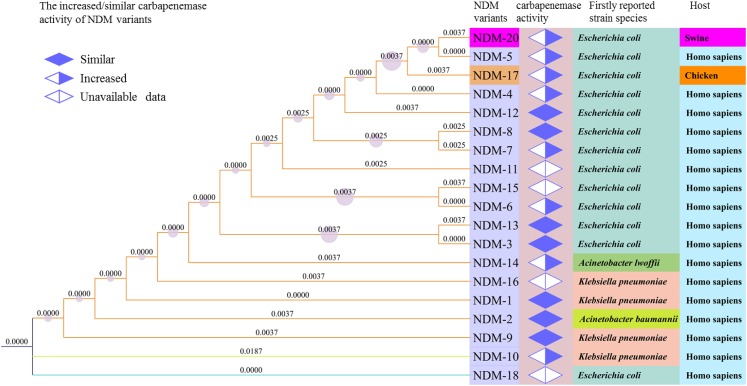
Phylogenetic relationships between New Delhi metallo-β-lactamase (NDM) variants based on amino acid sequences. The tree was generated in MEGA7 and iTOL using the maximum likelihood method. Increased/similar carbapenemase activity of NDM variants was determined from previously reported data and compared with NDM-1. The numbers on the branches signify represent branch lengths.

The *de novo* assembled genome of CCD1 showed that the number of contigs was 87 and length of N50 was 110,756 bp. PCR and sequencing analysis identified the presence of a novel NDM-encoding gene, designated *bla*_NDM-20_ (GenBank accession no. KY654092). Relative to *bla*_NDM-1_, *bla*_NDM-20_ contained three point mutations at positions 262 (G→T), 460 (A→C), and 809 (G→A), generating amino acid substitutions Val88Leu, Met154Leu, and Arg270His, respectively (**Supplementary Figure S1**). Homology and phylogenetic analysis of the amino acid sequences of all NDM variants showed that NDM-20 shared the closest evolutionary relationship with NDM-5 (**Figure 1**). Notably, only one difference (Arg270His) was observed between the two variants, with this being the first description of a substitution at residue 270. In addition, sequence analysis confirmed the presence of *ampC*, *bla*_CMY_ (aztreonam resistance), and *sul2* (sulfonamide resistance) in strain CCD1 (Seral et al., 2012). However, no other ESBL-encoding genes were detected.

MLST analysis classified *E. coli* CCD1 as ST1114, suggesting that *E. coli* ST1114 strains have the potential to become a reservoir for the *bla*_NDM-20-like_ gene. A previous study showed that ST1114 *E. coli* isolates are the second largest reservoir of *mcr-1* (Matamoros et al., 2017), while our previous study identified two *E. coli* ST1114 isolates from chickens that were positive for both *mcr-1* and *bla*_NDM-5_ (Wang et al., 2017).

### Transferability of *bla*_NDM-20_ and Plasmid Analysis

**FIGURE 2:**
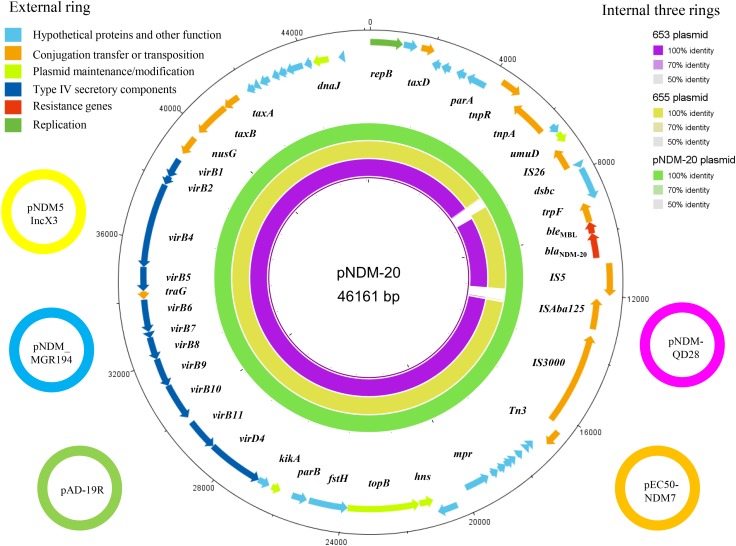
The five small external rings represent different *bla*_NDM_-harboring plasmids, shown in different colors that had >99% identity. The external ring represents the annotation of plasmid pNDM-20 (GenBank accession no. MF458176). Genes are color-coded depending on functional annotations. The internal three rings represent a comparative analysis of pNDM-20 (green) with two *bla*_NDM_-harboring plasmids, 653 (purple) and 655 (yellow) (constructed by BRIG).

Conjugation assays confirmed that *bla*_NDM-20_ could be transferred between *E. coli* strains, although the transfer frequency was only 5.01 × 10^-9^ per donor. Location analysis showed that the *bla*_NDM-20_ probe hybridized to a ∼47 kb plasmid band in the transconjugant DNA extracts (**Supplementary Figure S2**), indicating that *bla*_NDM-20_ was located on a transferable plasmid. Subsequently, the entire 46,161-bp *bla*_NDM-20_-harboring plasmid, named pNDM-20 (GenBank accession no. MF458176), was sequenced using a WGS approach (**Figure 2**). pNDM-20 was identified as an IncX3-type plasmid, a major type of plasmid mediating transmission of *bla*_NDM_ amongst CRE in China (Zhang et al., 2017a). In pNDM-20, *bla*_NDM-20_ was flanked in the upstream region by IS*3000*-ΔISA*ba125*-IS*5* and downstream by *ble*-*trpF*-*dsbC*-IS*26*-Δ*umuD*, a common *bla*_NDM_ genetic structure that will be responsible for horizontal transfer of *bla*_NDM-20_ among Enterobacteriaceae (Wang et al., 2014; Liu et al., 2017). No resistance genes other than *bla*_NDM-20_ and *ble*_MBL_ were identified on pNDM-20, supporting the observation that the transconjugants only exhibited resistance to β-lactams, and remained sensitive to aztreonam and other classes of antibiotics.

Homology analysis revealed 99% nucleotide sequence identity between pNDM-20 and IncX3-type plasmid pNDM-MGR194 (GenBank accession no. KF220657). pNDM-MGR194 was the first reported *bla*_NDM-5_-harboring plasmid, and suggested to be responsible for the evolution and distribution of *bla*_NDM-5_ amongst bacteria (He et al., 2016). To date, multiple pNDM-MGR194-like plasmids have been identified in bacteria isolated from both humans and animals in China. These plasmids which are 46,161–46,165-bp in length showed >99% nucleotide sequence identity to pNDM-MGR194, including pAD-19R (GenBank accession no. KX833071), pEC50-NDM7 (GenBank accession no. KX470735), pNDM-QD28 (GenBank accession no. KU167608), and pNDM5_IncX3 (GenBank accession no. KU761328) (**Figure 2**). Therefore, the identification of pNDM-20 provides further evidence that IncX3-type *bla*_NDM_-harboring plasmids have become established in bacterial strains originating from both humans and animals, and may circulate via the food chain (He et al., 2016; Liu et al., 2017).

Of note, pNDM-20 had the same backbone structure as the two *bla*_NDM-5_-harboring plasmids (653 and 655) from the ST1114 *E. coli* strains mentioned above. Further analysis showed that the two *bla*_NDM-5_-harboring plasmids could be typed as IncX3 using PlasmidFinder software^1^ (**Figure 2**). Given these plasmids have the same genetic background and plasmid group/type and were all isolated from ST1114 *E. coli* strains, it is possible that *bla*_NDM-20_ evolved from *bla*_NDM-5_ through a series of mutations.

### Functional Analysis of *bla*_NDM-20_

To determine the specific function of *bla*_NDM-20_, *bla*_NDM-20_, the reference gene *bla*_NDM-5_ (containing only a single nucleotide difference at position 809) and *bla*_NDM-1_ were cloned, and the corresponding transformants were examined to determine their antibiotic sensitivity profiles. As shown in **Table 1**, compared to DH5α+pHSG398, expression of *bla*_NDM-20_, *bla*_NDM-5_, and *bla*_NDM-1_ in *E. coli* DH5α reduced susceptibility to almost all tested β-lactams, including penicillins, cephalosporins, carbapenems, and combinations of penicillins and β-lactamase inhibitors, except aztreonam. In addition, the MICs of piperacillin was not affected by the addition of β-lactamase inhibitors tazobactam. In general, the *bla*_NDM-20_ transformant DH5α+pHSG398/NDM-20 exhibited similar β-lactam resistance to *bla*_NDM-5_ transformant DH5α+pHSG398/NDM-5, although several slight differences were observed. Compared with DH5α+pHSG398/NDM-1, the DH5α+pHSG398/NDM-20 exhibited a fourfold elevation in MIC of ertapenem, indicating that *bla*_NDM-20_ should enhance resistance to ertapenem. All our findings demonstrated that *bla*_NDM-20_ was not as effective as *bla*_NDM-5_, but conferred higher resistance to carbapenems than *bla*_NDM-1_.

### Enzyme Activity Analysis

To further characterize NDM-20 and explore the influence of the amino acid substitutions on enzymatic activity, NDM-20 and NDM-5 were successfully purified (>90%) and their kinetic parameters were examined. NDM-20 was able to hydrolyse all β-lactams tested, except aztreonam (**Table 2**). The *k*_cat_*/K_m_* ratios for ampicillin, cefotaxime, and cefoxitin for NDM-20 were higher than those for NDM-5, but carbapenems *k*_cat_*/K_m_* ratios for NDM-20 were lower than that of NDM-5. These findings resulted from the lower *K_m_* values for some penicillins and cephalosporins and higher values for carbapenems for NDM-20 compared with NDM-5. These results demonstrated that NDM-20 had higher enzymatic activity against some penicillins and cephalosporins and lower activity against carbapenems relative to NDM-5, caused by its enhanced affinity for some penicillins and cephalosporins and depressed affinity for carbapenems.

**Table 2:** Kinetic parameters of NDM-20 and NDM-5*^a^*.

β-Lactam	NDM-20*^b^*			NDM-5*^b^*			*k*_cat_/*K*_m_ (μM^-1^ s^-1^) ratio for NDM-20/NDM-5
	*K*_m_ (μM)	*k*_cat_ (s^-1^)*^b^*	*k*_cat_/*K*_m_ (μM^-1^ s^-1^)	*K*_m_ (μM)	*k*_cat_ (s^-1^)*^b^*	*k*_cat_/*K*_m_ (μM^-1^ s^-1^)	
Ampicillin	167 ± 1.9	92 ± 11	0.55	425 ± 27	103 ± 10	0.24	2.29
Amoxicillin	391 ± 35	15 ± 0.87	0.04	561 ± 17	25 ± 2.6	0.04	1.00
Penicillin G	313 ± 17	113 ± 2.1	0.36	283 ± 41	99 ± 5.5	0.35	1.03
Aztreonam	NH^c^	NH	NH	NH	NH	NH	NH
Cefotaxime	15 ± 0.18	18 ± 0.05	1.2	20 ± 0.89	19 ± 1.4	0.95	1.26
Cefoxitin	39 ± 0.34	11 ± 0.01	0.28	66 ± 3.3	11 ± 0.45	0.17	1.65
Ceftazdime	107 ± 8.1	19 ± 1.4	0.18	109 ± 12	19 ± 0.53	0.17	1.06
Ertapenem	278 ± 9.1	127 ± 5.5	0.46	124 ± 3.8	88 ± 3.2	0.71	0.65
Imipenem	377 ± 25	147 ± 3.3	0.39	302 ± 14	128 ± 1.2	0.42	0.93
Meropenem	362 ± 12	160 ± 4.9	0.44	257 ± 12	128 ± 0.65	0.50	0.88

^a^*The proteins were initially modified with a His-tag, which was removed after purification*.

^b^*K_m_ and k_cat_ values are means ± standard deviations from three independent experiments*.

^c^*NH, no hydrolysis was detected under conditions with substrate concentrations up to 1 mM and enzyme concentrations up to 700 nM*.

**FIGURE 3:**
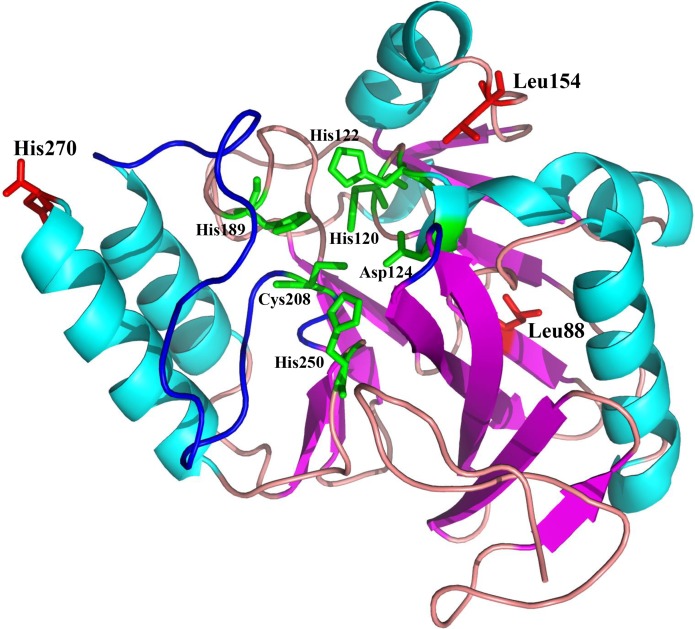
Homology model of NDM-20. Protein backbone of NDM-20, shown as a cartoon with the helices and strands. The three amino acid substitutions (L88, L154, and H270) are labeled and colored red.

NDM-20 contains an Arg270His substitution relative to NDM-5. This substitution may be responsible for the decreased carbapenemase activity of NDM-20 relative to NDM-5. Two other amino acid substitutions (Val88Leu and Met154Leu) were identified in NDM-20 compared with NDM-1. The Val88Leu substitution was reported to decrease the hydrolytic activity of NDM, while the Met154Leu substitution appeared to increase the carbapenemase activity (Nordmann et al., 2012). The active site of NDM-1 is formed by two loops, loop3 and loop10, and crucial amino acid residues binding to two zinc ions, including His120, His122, His189, His250, Cys208, and Asp124 (Green et al., 2011), and the residue 270 was located out of the enzyme active center (**Figure 3**). Based on this information, the substitution at residue 270 would not affect the conformation of enzyme active site. It is also possible that the Arg270His substitution may change the protein conformation of NDM-20, thereby altering its hydrolytic activity (Zou et al., 2015). However, further experiments are required to confirm which, if any, of these mechanisms contribute to the altered activity of NDM-20 compared with NDM-5.

## Conclusion

Our study reports a novel NDM variant, NDM-20, in a ST1114 *E. coli* isolate recovered from food animal in China. Compared to NDM-5, NDM-20 enhances the hydrolytic activity toward some penicillins and cephalosporins, but depresses the carbapenemase activity, which may affect drug strategies for CRE infection. Both the identification of NDM-20 from swine and NDM-17 from chicken suggest that the food animals have become the reservoir of bacteria producing NDM, which may drive NDM evolution to enhanced enzyme activity toward some or all β-lactams. Furthermore, *bla*_NDM-20_ positive bacteria possess the potential capability of dissemination in not only animals but human, becoming a worldwide spread risk. It is time to monitor bacteria producing NDM-20 and other NDM variants in food animals, and coordinate globally to prevent or slow down the NDM evolution.

## Author Contributions

JS was responsible for the study design. ZL, JL, XW, and DL assisted in data collection. ZL and YK completed the data interpretation. ZL, YW, and JS wrote the report. All authors revised, reviewed, and approved the final report.

## Conflict of Interest Statement

The authors declare that the research was conducted in the absence of any commercial or financial relationships that could be construed as a potential conflict of interest.

## Footnotes

1 https://cge.cbs.dtu.dk/services/

## Supplementary Material

FIGURE S1 Secondary structure and disorder prediction of NDM-20. Alignment was constructed in Phyre2 using NDM-1 as template (fold library id: c3rkjA in Phyre). The V88L, M154L, and R270H substitutions are boxed in blue.

FIGURE S2 Localization of bla_NDM-20_ on *Escherichia coli* plasmid pNDM-20 by Southern blot hybridization using a bla_NDM-20_ probe **(A)** and by S1-PFGE **(B)**. Lanes 1 and 3, bla_NDM-20_ transconjugant *E. coli* 20J3; lanes 2 and 4, *E. coli* J53; lane M, marker, *Salmonella* H9812.

DATA 1 The sequence of bla NDM-20 gene has been deposited in GenBank under accession no. KY654092. The sequence of bla NDM-20 gene as follows:

DATA 2 The complete nucleotide sequence of plasmid pNDM-20 has been deposited as GenBank accession no. MF458176. The sequence of plasmid pNDM-20 as follows:

TABLE S1 Amplified and cloning primers.
