# Corrigendum: Novel Variant of New Delhi Metallo-β-lactamase, NDM-20, in *Escherichia coli*

**DOI:** 10.3389/fmicb.2018.00497

**Published:** 2018-03-23

**Authors:** Zhihai Liu, Jiyun Li, Xiaoming Wang, Dejun Liu, Yuebin Ke, Yang Wang, Jianzhong Shen

**Affiliations:** ^1^Beijing Key Laboratory of Detection Technology for Animal-Derived Food Safety, College of Veterinary Medicine, China Agricultural University, Beijing, China; ^2^Key Laboratory of Genetics & Molecular Medicine of Shenzhen, Shenzhen Center for Disease Control and Prevention, Shenzhen, China

**Keywords:** NDM-20, carbapenemase, ST1114, IncX3, kinetic parameters

There was a mistake in the grant number of the funding as published. The correct version of funding appears below. “The study was supported by grants from the National Natural Science Foundation of China (31530076, 31422055, and 81661138002) and the National Basic Research Program of China (2013CB127200).” The authors apologize for the mistake. This error does not change the scientific conclusions of the article in any way.

The original article has been updated.

## Conflict of interest statement

The authors declare that the research was conducted in the absence of any commercial or financial relationships that could be construed as a potential conflict of interest.

