# Supplementary figures and images for: Novel Variant of New Delhi Metallo-β-lactamase, NDM-20, in Escherichia coli
Source: Front Microbiol. 2018 Feb 21;9:248. doi: 10.3389/fmicb.2018.00248 (PMC5826333; doi:10.3389/fmicb.2018.00248)

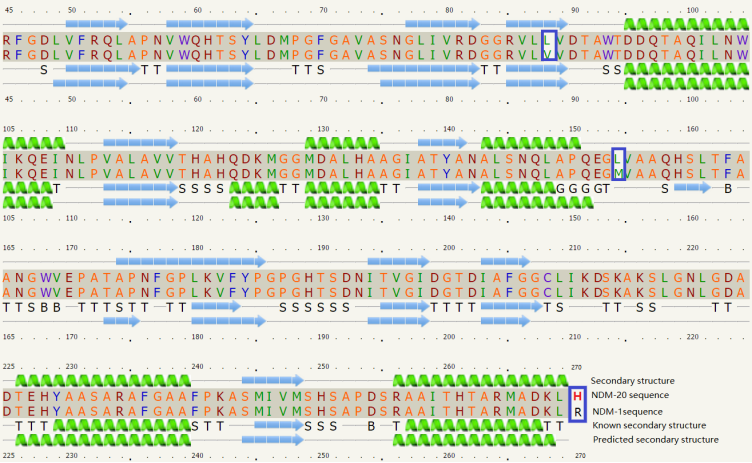

Supplement: FIGURE S1 — Secondary structure and disorder prediction of NDM-20. Alignment was constructed in Phyre2 using NDM-1 as template (fold library id: c3rkjA in Phyre). The V88L, M154L, and R270H substitutions are boxed in blue. [file Image_1.TIF]

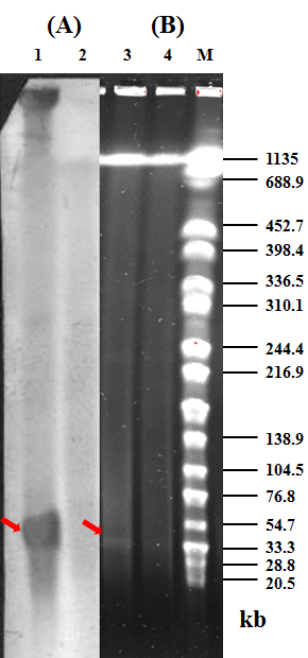

Supplement: FIGURE S2 — Localization of blaNDM-20 on Escherichia coli plasmid pNDM-20 by Southern blot hybridization using a blaNDM-20 probe (A) and by S1-PFGE (B). Lanes 1 and 3, blaNDM-20 transconjugant E. coli 20J3; lanes 2 and 4, E. coli J53; lane M, marker, Salmonella H9812. [file Image_2.TIF]
