# Supplementary material for: Novel Variant of New Delhi Metallo-β-lactamase, NDM-20, in Escherichia coli
Source: Front Microbiol. 2018 Feb 21;9:248. doi: 10.3389/fmicb.2018.00248 (PMC5826333; doi:10.3389/fmicb.2018.00248)
Supplement: DATA 1 — The sequence of bla NDM-20 gene has been deposited in GenBank under accession no. KY654092. The sequence of bla NDM-20 gene as follows: [file Data_Sheet_1.DOCX]

**The sequence of *bla*_NDM-20_ gene has been deposited in GenBank under accession no. KY654092. The sequence of *bla*_NDM-20_ gene as follows:**

ATGGAATTGCCCAATATTATGCACCCGGTCGCGAAGCTGAGCACCGCATTAGCCGCTGCATTGATGCTGAGCGGGTGCATGCCCGGTGAAATCCGCCCGACGATTGGCCAGCAAATGGAAACTGGCGACCAACGGTTTGGCGATCTGGTTTTCCGCCAGCTCGCACCGAATGTCTGGCAGCACACTTCCTATCTCGACATGCCGGGTTTCGGGGCAGTCGCTTCCAACGGTTTGATCGTCAGGGATGGCGGCCGCGTGCTGTTGGTCGATACCGCCTGGACCGATGACCAGACCGCCCAGATCCTCAACTGGATCAAGCAGGAGATCAACCTGCCGGTCGCGCTGGCGGTGGTGACTCACGCGCATCAGGACAAGATGGGCGGTATGGACGCGCTGCATGCGGCGGGGATTGCGACTTATGCCAATGCGTTGTCGAACCAGCTTGCCCCGCAAGAGGGGCTGGTTGCGGCGCAACACAGCCTGACTTTCGCCGCCAATGGCTGGGTCGAACCAGCAACCGCGCCCAACTTTGGCCCGCTCAAGGTATTTTACCCCGGCCCCGGCCACACCAGTGACAATATCACCGTTGGGATCGACGGCACCGACATCGCTTTTGGTGGCTGCCTGATCAAGGACAGCAAGGCCAAGTCGCTCGGCAATCTCGGTGATGCCGACACTGAGCACTACGCCGCGTCAGCGCGCGCGTTTGGTGCGGCGTTCCCCAAGGCCAGCATGATCGTGATGAGCCATTCCGCCCCCGATAGCCGCGCCGCAATCACTCATACGGCCCGCATGGCCGACAAGCTGCACTGA
