# Supplementary material for: Novel Variant of New Delhi Metallo-β-lactamase, NDM-20, in Escherichia coli
Source: Front Microbiol. 2018 Feb 21;9:248. doi: 10.3389/fmicb.2018.00248 (PMC5826333; doi:10.3389/fmicb.2018.00248)
Supplement: DATA 2 — The complete nucleotide sequence of plasmid pNDM-20 has been deposited as GenBank accession no. MF458176. The sequence of plasmid pNDM-20 as follows: [file Data_Sheet_2.DOCX]

**The complete nucleotide sequence of plasmid pNDM-20 has been deposited as GenBank accession no. MF458176. The sequence of plasmid pNDM-20 as follows:**

ATGCGGTTGTTGCTATCTTTAGATATGAAGATCCTCAGATCTTCATATCTAAAGGTGAGAGGTTTTTTAATTAAAGGTTGTATTGTTGTCTTGAATTACAACCTTTGTGGGGTTATGATTTGCCTACATAGGAAAGGTTATATGAGGCTTATCGTGAAGACAGTAACGGGATTAACGAAAGTTAGACATAGAAATGAAGTTGGGGTAACTCTTGCATCCCTTTCCCTTTCAGCAAAAAGAGTGCTTTTTCTGGCTCTTTGCCAGATTGATACAAAGGAAATGTTAGATGATGATATTTTGGAGGTTGATGCTGACTTTTTTTCAAAAGCTACTTCTTTAGATAAATATGCCTCTTATGCAGCTCTGAAAGAGGGAGCTAAAGTTCTTTCTTCAACTACGTTAGTTTTAAACAGAGATGATTTAAAAAATTTGGCTGATGAACTTGGGATACTATCATCTAAGAATAAAATACCTGATCGCTTGGATTTAAACTTAACAGAATTCTGTGCCTATTACGATCATCTTGCAACTGTACGGATAAAATTCACAAATACAGCTAAGCGATATTTTTCTAAGTTGATTGGTTCTGAAAATAGATATACCACTCAGGTATTGAAATCAGTTGTGTTGTTGAACAGTGTAAACTCAACAAACCTTTATCAAGTCATTAGGAAATATTACAGTCAGAATAGTTCAAAAAAATCTTTTGATATATCCGTGGATGATTTAAAAGAGGAAATGGGACTTTATACTATTGAGGAAGGGGAGAAGAAATATAAGTATCCTAAATACTCATTTTTTGTTCGTGATGTTATCAATAAAAGCATTAATGAGATAATTGAGAAAACGGAAATAAATCAGTTATCTTTCTCTGTTGTTGGGAAAAAAGGGAGAATGGCTCACATGCTTAGATTTGAGTTTAGTATAAATGAAAAATCCTCATCCTTTTCGGAAGATGATATGGCATTTTTAGAGGAGTTTGACAAAGTCGTTCCACCAAAAAAGAATAAGTGAGGCAAAACATGGCTAAAATATATGAGTTTCCGCAGGGAGCCGAACGAAGTAAGCTGAAAAAAGAAATCATCCGGGAACGTAAAAAACGATTGCGAGAAACGAACGGTAACCCGGTTATCCGGCACGCTAAATGGTTCTGGTTTTATCTACGTCTTGCTACGGCTGGCGCACTGCATTTAGTCTCTGTTATTTCTCTGGCAGTCCTGGGGGCTTTCAGTAAAGCTATTTTCTGGATTGGTGGAATGCTCTGTGTTGTTACATGGTTCCACCTTGAGCGCCAGTTCTGGACGCCGCAAAATTTCACGATTCCAGTAATCGTAACGCTGTGGGGATTGAGTTTGTTTGCCACTCCCCTGATGGAGCTGCTTAACAAAAAAATGCCGTGGTATCGTCTTCTGGTTCCTGATGCAAAGCACACTAGCACTGAGGCAACCAATGATGATCAGCCGTGAAGCAACAATGTTATCCAGTCGGCTTGCAGTGCTTCTTTCAGCCTTTTATCATCGGTTAATCATAGCGCACGAGGCGAGTTCTTTTCACTGCGATGCAGGATAGGGCAAACGCCGCAAAATGACGTCTCTGACGCCATTCCGCAACGTTTGCCCGGTGACCTACAGTCGGTTCTTGTTGGAGAGAGTTATGAAATATCAGGTTAAAGAGTTTATTAACGAAAAATATTCTAAGGCTGTTAATATTTTAAAGGATAACCTTAAAGAACACTACCATATTTTTTATGGTTTGAGATTAAGTGAGATTCTTTTTCCTGCCAGCGAATACGGCTCTGAAATGTTTTTTCAGGAGTTTGAAGCGATTAACAGTGTAATATTGCCTTTGGTGATTTTTGATTTGATAGACCGAAAGCCGATTATGGTTATCGGCTTCGGTGAAGTATGTGGAGTTGATTCACTTGTTGATTCTGGTATAGAAGTTGTTTCTTTAGACGGTTTAAGCGATCTGCTACTGGTTGAAAAACTGACGCCATTATTTAACTAAACCTCTGGATAACTATCTTTTCAGTTTTCCAGTAAGATTTTTTCTATATCTGAAAGCACTATTTTTTCAATGCTTTCTATATAGTAATGCTCATTAAAACCTGTCATTTCTGTAAATTTTAGCTCTGCATCATCTTTCGAATAAGCAATAACAACTCTGAAATCATTTTCCCACAAATCCCACCTGTATTTGTCTTTTATACGAACCCTGTACAATCCTTTTTGGATAGTGCCATCTTTTTTCTCTTCCTGTACTTTTTTTAATTTTTCATTTTGCACCTGTTGTTCAAGCCATGATTTATTCATAAATCCCCCACGGTTAACGTCTTTAGGCTCGGTTTTTACTTAACGAAATCTTCCGGTTTTACACCTGTTTTTACCTTCATATACTTGCCCTCCCCCCTTAGCTTCGGCTGAAGTTCGGTAAAGTACCATCCGGCAACGGTAATAATGCCTAAAAATACAAAAATAGCAGGCTTCAGAACATCAATAGCTGATACAGCGCATAACTCAATATTGAGATTAGTACAAGTTCCCTCATCAGGTGCATAGAACCCCCATAAGCCTATACCTATAAGGAACATCCCCAATATCGGTTGGATGTATAACAAGTAGATATTAATAAGAGTTTTAATATATCTGAATATGAACATTTTTCTTTCCTTAGGTTTCTTTCTATTTTGGATTTCCAATTCTTAACCAATGTGATGGTTCGACAATCCATTCTCCGCAGTCATAGGCAACGGCCTTATTATGCCTGTTAGCGAACCATACTAACCAGGCTAATAATCGCTGATTGCCGCTGTATTTGGTTTATCTCTAACCATCACAACATATCTCTTTTTCTTAGCCATAACGCTACCCTAATTTTGCAGATTCAAGCAGCCAGTGGTTTTTCAGGTTCTCGTCCAGGTTATCGAATGCGGCCAGCGCTGCCTTTAGAACGGTTGTCTTGCTCTGGCCTGTTCTCTGCGCCTCCGCTTCCAGGATCTCCTCAAAGTACGCTGGCAGTCTGAAGTTCATCAACTTTGGTTTACCCGCTACAGGCGCTCTGGTAGCGCCGGAGATAAAACGCGCGGTTTCTGCGTTTGTTGCTGGTTGCTGAGATTCATCGATGTTCGGGCGGTTTAGTTTTAAAGCCATGAAAATTTCCCCTTGTACGGTATGCATTAATTGTAAATATCATAGTATCTTTATGGTGTATTTACAATGTAAAGATTTCGATGAATATTGTTGATATTTCTATAAAATGCCAATTTCCTGTGCCACAAGTTCAATCTGTGCTTTCGCTGTTGAAAGGCTCGATGCCCGGCTAACGTCATGTACCCCTGCCCCTTCATTACAGGCGCTTTCATACACATCCAGCTCGGATATCCGGGTTTTCAGAGGTTGAATCCAGATGTTGTCCGAACGCAGTAATTTATCCAGATCAATGGCCGCTTTTCTGTGGCGGGGTTTCGCTGAGTTGATTCTGGTGAACAGCACCCACGGTTCCAGAGCTGCGTTAGCTTTCTGAGCGGTGCGGATTTTCTCTGTAACACTGGTCAGTGTTTCGCTTTCGAAATCAGAGGACGGCTTAACCAGTGTCACCACGATATCTGATACTGTCAGGGCGCTACGGAACTCCTGGCTGTCGTGGCCGGGGCAATCGACAATGAGAACTTCACACAGCTTACTAAGGCGCTTAATTTCGTTGCTGACGTTTCCGTAAGCTGCGTGAACCGGGATAGTGAGTAAGCCGTTATCTGTCCGCTTTTCGTTCCAGCTCAACAGATCGTGGTTTTTATCTGTCTTCAGGATAATTACCGACTTACCTTTGTTGACCAGCATTGAGCCTGTGTTAGCCACGTAGGTGCTTTTGCCTACCCCACCTTTATCTGATACGACAAGCAAGATTTTACCCATTTCAGAATCCCTCTCTTTATGTTGTAAAGCTCATGTAAAGATATGAATGTCTTTACATGGTGTGTTTTTCGTAAAGATACTTTTGTCTTTACAGTGTCTTTATAATGCAATGGCTTTACCATGTAAAGCACAAATAAAGATATATTTATCTTTATGGTGTCTTTACATGGTAAATGTTGCTGTGTCTTTATGTGCGCTTTATGGTGTAAATCTTGGTGTGTCTTTACATCATCTTTATGTTGTAAATTTTTAGTGTGACGTTGTCACTATAGGGCTGGGGTCTGTTTGCTGGTTCTTTTTGAGTGTGCCTTCCCTGAACCAACTGAAATGGGTACTGTATGGGTGTGTCTGGTTAACTCAAAATGAACTGGTTCCCTATAATGACTCAAACAGTACCGAAAGGCTTTGTCAGAGCTTACTTACGCGCATCCACGACTGAACAGGATGCAACACGCGCCCTGGACACGATAAATACCTTCGCCATCGAGCGCGGCCTGAGCATCTGTAACTACTACATTGAAAATGAATCAGGTTCTAAGCTTGAACGCCCTGAGTTGTTCCGGCTCCTGAAAGATTGTCAGCAAAACGATATCTTGCTGGTAGAGGATGTGGACAGGCTTTCACGCCTGGTAGGGGAGGACTGGAACACACTGAAGAAAATGATCCGGCAGAAAGATATCCGGGTTGTGGCCGTCAACGTTCCGACAACCTGGCTCGCATCGGGTCACAATGATTTTGACAGCCGAATGTTTTCGGCCATAAACGATATGTTGCTGGATATGCTGGCCGCCGTCGCCAGGCGAGACTATGAACAGCGCCGGGAACGGCAGCAGCAGGGGATCGCCAGGGCGAAAAGGGAAGGGAAGTATAAAGGCCGCCAGGTTAATCAGTCTCGCTATGACGCCATAAACAGGCTGATTGCGAGCGGCAGCTCCTGGAGCCAGATTCAAAAAGTGCTGGGTTGTAGCCGGGCGACTATCAGCAAAGCTGTGAAGCAACAGACCGTTAACAAAATGAACCATGAACCTGAAGGTAGGGAGTAGTTACAACATAATAAACAGTGTGTTGAAGAATAAAACTGATTCTTTATACTGTATATGCATGGGTCTTCCCCGATCATGGTGGGAAGGCTCAGAACGCCATATTCAGCTTTCCGTAGTGGAACATCACCCCCAGTTTAAAGCGCTCCCGGTTTCGGTATCCCCTGGCTTTTATCCTCAGCAGCCTGATCTTGCTGTTAAGTGCCTCCGCATTTCCGTTTGAGACACTGTGTCGCATCGCATTCAGGATCCCGTACAGCCTTTTTCCTATCGTTTTCGCGGCATTTTTCATCATGGGAACGTCACTGTTAGCCGCCAGCGCCAACCATCTCTGCCAGTCACTCCGTCTTTCCTCGCTCCATGGCCTGTTCCAGATATCCTTTGCCAGCTCTTTCAGCGCCCAGCACTGGCTCGTCAGCTTCATCTGTGCACGCAGCCACATCAGCTTTTCCTGCCGGGATTCGGTCATCCACTTATCGCTGTACTGCCACAGGAAGCGGGTTCCTTTTGCCTGGTGTCGGCTTTCAACAGGGAGGTGCGGATGTTCATTCTGACGGGTTTTATCAACTACCTCGCCCAGTTGCTTCGCCACATGGAAGCGGTCAAAGGCGATTTTCTCAACCGCACTGGGTAAGTGGATACGCGCTGCTCTTATATAGCCCGCGTTCATGTCCATTGAGAGCGTTTTGATAGCCAGCAACTGCCCATCAGTGAGCGTGCGAAGATAGCCGGCAAGACTCTCTGTGCCGCGATCATCCGTTAAGGCCAGCGCCCGACCATCGCGATCGGAGATCACCGTTATGTAACGATGTCCTTTTTTAAAGGCGACCTCATCCACATTCATATGACGGGCGGATAATGGCTTTTTTATCCGGGCAAGACCTCGCTTAACTGCCCGGGTCATAATGCCGTCAACCGCATTCCAACTGAGCTTAAGTTGCTTCCTGACAGCATCAACGGTGCTGATTTTCAGCCATGAGAGAACGAACGATTCGAATAGCAACGTATACCGGCTTCCGGGGCCAGCCCACGGAACAGGCAACGTCAGGCAGCCATGCTCCGGACACATAATTCGTGGAACATCGGCTTCAACAATAGTGGTGAACTGGCAGGTATCAAGATGGCGCCATTTACGATGACGGTGATCGTGAACAGAACAGGATTTACCGCAGGTCGGACAGGCTAGCCGGGTGTTTTCAGCGATCTCAATAGTGACAGTAACAGAACCGGCATTTTCATCGAGAGAAAGGGACTTTACCTGCCACGGATCGGACAGGTTGAGAATATGAGCGTAGAGGGACTTTTCGTCCATGGCGGTGACCTCTGGCGATTAAATACACCATTATCATGCCTTCAGCCACCACAACAAGGGAAGACCCATATGCATACAGCTATATTGATCGGAGGTTTTTCATGCCTAGACACTATGAAATTGACTCAGCCTGGCGTGCGAGCATTAAGCGTGAGCCGAACGGTCGTCAGACAGTTACGACAGAAGCCTTTGTCAGCCAGCTTGCGCTGATTAATTTTCACTGGAGTTGTCGCCAAGCAAATCAGTGGATTGAAACCTACGTGACCGTTTTTAAAGACATTTCCACTCAGGAAGGAGAGAACCGGACATTCATGCTTTTTAACCCCAACGGAGGCCGTTAAAAATGGGTTTTCCATCGCCAGCAACCGACTACCAGGAAAACAGGCTGTCACTCGATCAGCACTGTAATACAGGAGCGCCGGGAGTATTCTTTTTTCGAAGTGATACCTACTCTTTCCGGGAAGGGATAAAGCCTGGTGCGCTGCTCATAGTAGATTTTGGGGGCACGCCCGTTGACGGGTCTTTGGTGCTCTGTGTCCTGGAACAAGAATTCAGGATCATGCGATTGCGACTACACCCCAAGCGGTGTCTCCAGGAACTCGATAAGCTGGATAACTTCAGGGCTATACCTGATGACGATGAGGACGGGTTGGAGGTCAGGGGGGTCATTACGCACATTGTGGCACTGTTGCAAAGTTAGCGATGAGGCAGCCTTTTGTCTTATTCAAAGGCCTTACATTTCAAAAACTCTGCTTACCAGGCGCATTTCGCCCAGGGGATCACCATAATAAAATGCTGAGGCCTGGCCTTTGCGTAGTGCACGCATCACCTCAATACCTTTGATGGTGGCGTAAGCCGTCTTCATGGATTTAAATCCCAGCGTGGCGCCGATTATCCGTTTCAGTTTGCCATGATCGCATTCAATCACGTTGTTCCGGTACTTAATCTGTCGGTGTTCAACGTCAGACGGGCACCGGCCTTCGCGTTTGAGCAGAGCAAGCGCGCGACCATAGGCGGGCGCTTTATCCGTGTTGATGAATCGCGGGATCTGCCACTTCTTCACGTTGTTGAGGATTTTACCCAGAAACCGGTATGCAGCTTTGCTGTTACGACGGGAGGAGATATAAAAATCGACAGTGCGGCCCCGGCTGTCGACGGCCCGGTACAGATACGCCCAGCGGCCATTGACCTTCACGTAGGTTTCATCCATGTGCCACGGGCAAAGATCGGAAGGGTTACGCCAGTACCAGCGCAGCCGTTTTTCCATTTCAGGCGCATAACGCTGAACCCAGCGGTAAATCGTGGAGTGATCGACATTCACTCCGCGTTCAGCCAGCATCTCCTGCAGCTCACGGTAACTGATGCCGTATTTGCAGTACCAGCGTACGGCCCACAGAATGATGTCACGCTGAAAATGCCGGCCTTTGAATGGGTTCATGTGCAGCTCCATCAGCAAAAGGGGATGATAAGTTTATCACCACCGACTATTTGCAACAGTGCCCCCCGCCCTGCAGGAACGCCTGCGCCAACTGCATCCCTACGAACTCCCGGAGCTGCTCGCGGTCGAAGCCGCGTCCGGCCTGCCCGAATACCTGCAATGGCTGGCCGCCGAGAGCCGACCGGTAAACTGAGCCAATGACCGCATCCACGATCCGCCTGCGCCGCTGGCTGGCCGGGCTCGCCCTGCTGCTTGCGCTGCCCGCGACCTCGGCCGTCGCCCAGGACTTCGAACTGCCGCCGGTCGACGAGGTCTTCGTCCTGTCCGCGCAGGCCACCGCGCCGGACCGCATCGAGGTGCGCTGGCGGATCGCCGACGGCTATTACCTGTACCGGCACCGCACCTCGGTCAAGGCCGATGCCGCCTTCACCGGCGCGACCATGGCGCTGCCGAAGGGCAAGGCCTACCGCGACGAATTCTTCGGCGACGTCGAAACCTACCGCAAGGAATTGCTCGGCACCCTCACCGGCACGCCCGCGGCCGGCGCGAGCGCGACCACCCTGACCGTGAAGTACCAGGGCTGCGCCGATGCCGGCGTGTGCTACCCGCCGCAGACCCGCACCCTGAAGGTCGCGTTGCCGGGCGAAGCGGGCGCTGGCGGCTTCGGCTGTAAGGCGCGCGGGCTGCATGATTATGTCGTCAAGAACGGCTCTGCCGATCATCCCAACGCCGAGGTGAAATTCGCACTGGGTGATGTGGTCAACACCATGATCGGCTGCACTAATGGTGAAACGATCATGCTGTGCCACGACACCTCGCTGCCGCGCCCCTATTCTCTCGGCTTTCGGGTGCAAGGCACCGAGGGGCTGTGGATGGACGTCAACAAGTCGATCTATCTGGAGGGCAAGAGCCCACAGCCGCACCGCTGGGAGCCTGCCGAGGGCTGGTTTGCGAAATACGATCACCCGCTATGGAAACGCTACGCCGATCTGGCGGCAGGGGCCGGGCATGGCGGGATGGACTGGTTCGTGATCCACGCTTTTGTCGAGGCGCTGAAGGCCAAGGCCCCGATGCCAATCGACATTTACGACGCGCTGGCCTGGAGCGCGATCACGCCTCTGTCGGAACAATCGATTGCTGAGGGCAATCGCACGTTAGATTTTCCCGACTTCACCCGAGGGCAGTGGCGCACCCGCAAGCCGATCTTTGCGCTGAACGACGCCTATTGATCGACGCGATTTAGGCCAAGCGCACCGCAAAGGCGAAATTGGTAATCTTGTCGGTATCCTTGACGCCCGGCGCGCTTTCGACGCCGCTGGAGGTATCGACCAGCGGCGCTCCGGTGCGCGCAATCGCCTCGGCAACATTCGTCGGATTTAGCCCGCCTGCCAGCCCCCACGGCAAGGCACCGCGATATCCGGCCAGCAGCGACCAGTCGAACGCCAACCCCATGCCGCCGGGCAGCGCGCCTTTGGGGGTCTTGGCGTCGAACAAGATCAAGTCCGCCGCCCCGGCATAGGCTGCGGCGCGTGCGACATCGCTGGCGCTGGCGACGGGCAGCGCCTTCCACACCGGCTTGCCAAACCGCGCGCGCAACTGGGCCACGCGTTCGGGCGATTCCGAACCGTGCAGCTGCAGCGCGTTCAGCTTGGCTGCCACCAGTGCGTCGGCGATGACAGCATCATCCGCATCGACGAACAAACCGACCATGGCGATCTGGCCAGCTGCGCGCGATGTCAAAGCGCCCGCGACATTCGACGTAACCGCACGGGGCGACGCTGGATAGAACACCAACCCGGCATAGTCCGCCCGCGCCGCGATGGTCGCATCGAGCGCCTCGGGTGTGCTGATCCCGCAAATCTTGATTTTCGCGGGCATGCGGTCAGTCGGGGTTCTGGATCAGCCGCACCAGCGTGCAGTCGGGATCGATCAGGTAGCCGATCCTCAGGCCGCTCGCCTCCAGTTGCGGAGCTTTGAAGCGCGGCCAGCCGGTGCTTTTTTCCTCGGCTCCCGCCGCGTTCACCAATGCCACCATGGCATCGAGATCATCCAACCGCAGGCAACAGCCGAACGAGCTCGTAGCTGGGTCGAGGTCAGGATAGGGGAAGAATTCGAGCTGCAAACCGCCGCGCTGCAGGATCATCCAGCCGCGATCCTTCCAACTCGTCGCAAAGCCCAGCTTCGCATAAAACGCCTCTGTCACATCGAAATCGCGCGATGGCAGATTGGGGGTGACGTGGTCAGCCATGGC**TCAGTGCAGCTTGTCGGCCATGCGGGCCGTATGAGTGATTGCGGCGCGGCTATCGGGGGCGGAATGGCTCATCACGATCATGCTGGCCTTGGGGAACGCCGCACCAAACGCGCGCGCTGACGCGGCGTAGTGCTCAGTGTCGGCATCACCGAGATTGCCGAGCGACTTGGCCTTGCTGTCCTTGATCAGGCAGCCACCAAAAGCGATGTCGGTGCCGTCGATCCCAACGGTGATATTGTCACTGGTGTGGCCGGGGCCGGGGTAAAATACCTTGAGCGGGCCAAAGTTGGGCGCGGTTGCTGGTTCGACCCAGCCATTGGCGGCGAAAGTCAGGCTGTGTTGCGCCGCAACCAGCCCCTCTTGCGGGGCAAGCTGGTTCGACAACGCATTGGCATAAGTCGCAATCCCCGCCGCATGCAGCGCGTCCATACCGCCCATCTTGTCCTGATGCGCGTGAGTCACCACCGCCAGCGCGACCGGCAGGTTGATCTCCTGCTTGATCCAGTTGAGGATCTGGGCGGTCTGGTCATCGGTCCAGGCGGTATCGACCAACAGCACGCGGCCGCCATCCCTGACGATCAAACCGTTGGAAGCGACTGCCCCGAAACCCGGCATGTCGAGATAGGAAGTGTGCTGCCAGACATTCGGTGCGAGCTGGCGGAAAACCAGATCGCCAAACCGTTGGTCGCCAGTTTCCATTTGCTGGCCAATCGTCGGGCGGATTTCACCGGGCATGCACCCGCTCAGCATCAATGCAGCGGCTAATGCGGTGCTCAGCTTCGCGACCGGGTGCATAATATTGGGCAATTCCAT**CAAGTTTTCCTTTTATTCAGCATTAAAAACCCCGCAAATGCGAGGCCTAGTAAATAGATGATCTTAATTTGGTTCACTGTAGCAAAAATATGGGGCGAATTCAAACATGAGGTGCGACAGTTTCAAAAGCCATATGATAATCAACAAGCTGAGCAAATTTCTCTAAGGAAGGTGCGAACAAGTCCCTGATATGAGATCATGTTTGTCATCTGGAGCCATGGAACAGGGTTCATCATGAGTCATCAACTTACCTTCGCCGACAGTGAATTCAGCAGTAAGCGCCGTCAGACCAGAAAAGAGATTTTCTTGTCCCGCATGGAGCAGATTCTGCCATGGCAAAACATGGTGGAAGTCATCGAGCCGTTTTACCCCAAGGCTGGTAATGGCCGGCGACCTTATCCGCTGGAAACCATGCTACGCATTCACTGCATGCAGCATTGGTACAACCTGAGCGATGGCGCGATGGAAGATGCTCTGTACGAAATCGCCTCCATGCGTCTGTTTGCCCGGTTATCCCTGGATAGCGCCTTGCCTGACCGCACCACCATCATGAATTTCCGCCACCTGCTGGAGCAGCATCAACTGGCCCGCCAATTGTTCAAGACCATCAATCGCTGGCTGGCCGAAGCAGGCGTCATGATGACTCAAGGCACCTTGGTCGATGCCACCATCATTGAGGCACCCAGCTCGACCAAGAACAAAGAGCAGCAACGCGATCCGGAGATGCATCAGACCAAGAAAGGCAATCAATGGCACTTTGGCATGAAGGCCCACATTGGTGTCGATGCCAAGAGTGGCCTGACCCACAGCCTAGTCACCACCGCGGCCAACGAGCATGACCTCAATCAGCTGGGTAATCTGCTGCATGGAGAGGAGCAATTTGTCTCAGCCGATGCCGGCTACCAAGGGGCGCCACAGCGCGAGGAGCTGGCCGAGGTGGATGTGGACTGGCTGATCGCCGAGCGCCCCGGCAAGGTAAGAACCTTGAAACAGCATCCACGCAAGAACAAAACGGCCATCAACATCGAATACATGAAAGCCAGCATCCGGGCCAAGGTGGAGCACCCATTTCGCATCATCAAGCGACAGTTCGGCTTCGTGAAAGCCAGATACAAGGGGTTGCTGAAAAACGATAACCAACTGGCGATGTTATTCACGCTGGCCAACCTGTTTCGGGCGGACCAAATGATACGTCAGTGGGAGAGATCTCACTAAAAACTGGGGATAACGCCTTAAATGGCGAAGAAACGGTCTAAATAGGCTGATTCAAGGCATTTACGGGAGAAAAAATCGGCTCAAACATGAAGAAATGAAATGACTGAGTCAGCCGAGAAGAATTTCCCCGCTTATTCGCACCTTCCCTAATGGTGTAAGCCAATCTAACGCCTTTCTAGGACGAGTATTCAGTGACATGGCAACTTGATTTAAATAATGCTGATCTGCCTGATTTAAATCAATCCCTTTAGGTAAATATTGCCTAATTAAACCATTCATATTTTCGCATGTGCCTTTTTGCCAGGGTGAATGTGGGTCACAGAAATATACATCTATGCCTAAATCTTCTTCGAGTATTTTATGTTCTGACATCTCACGTCCACGGTCATAGGTCAACGTTTTACGCAGTTCTGCAGGTAAATATTTCAGAGCTTCAGTTAAAGCCTTGCGCACTGATTCTGCCTTTGCATCAGGTAATGTTGCCAAGATACAGAGCCGTGTATTTCGTTCAATAAGTGTTGCTATCGAACTTTTATTGTCTTTACCTTTAATTAAATCAGCTTCCCAATGACCCGGTATTTTTCTTTCTTGAACTTCGGCTGGGCGCTCATGAATAGTTTTAATATCCTGTAATATAGAATCTTTTTTAGGTTCACCGTTAGCTTTTCGCTTTTTATTTTCATGACGTAGACAGGATAATAAGTCTTTTTTCAACTCACCCTTTGGTAATGCTCGTATCGTTGAATAAATCGTTGTATGGCTTACATTCATTGTTTGATCCAAATCAGGAAATGTCTTTAAACGCTTTGCTATTTGCTGAGGAGACCATAAACAACGGATCGCTTCAACAATAAATTTCCAGAGGATTGAATCGATTTTGAGTTTTCTGTGACCACGTCTACGTCTAGCGAAGGTGTTATCAGAAGCATATCGAGCTTGATAAACGTCATTGATGCTATTTCTTTTAAGCTCACGATAGATCGTACTAGGATGTCTTTTAATGAGTTCAGCAAATTTTCTGGCTGAAAAGCCTTCTTTTCTTGACTCAAGCATTAATGCAGTACGATCTTCAAAGTTAAGATGATGGTATGACAATTTTATATACTCCATAAACCCTTTAAATTAATTAGGTGGTTTATGTCGCACTTCAAGTTTTACTCTGCCAACTTTAAATCAGTGGCTCCACTAACACTCTTCACTTATTTATTTAAAATTTAGCGACGTTAAAAGACAATTAAGTATTTTTAGATAATGGGGTTCTAGGGATTTTCCGTCCAAAAACGACAAAGTGCTCTGGAGGCCGCGCCGTCCGTGGCCTCCAGAGGGGTATTACTTTTCGCTGACGGGTAAATATCCCTCGATTGAGCGCATAAGCTCCTCCAGATCTGGCAATTTTTCGCTCAATTCGAAGTGGTAAGTCCCCTTGAGGTTAATGTTGTGCCAGGCCACAGGGGATGCCTGTTTGACGATATCTATTCTCTTGGTATCTCCTTGGTATTCGAAGCTGGTCAACAGCTGGCTGAGTATCCTGGAGTTGAAGTAAACGATGGCATTGGTGACCAGGCGAGCGCACTCATTCCATAGCTGGATTTCTTCGTCTGAACTGCCCCGGAACTGATCCCCATTGACGCTGCTCACGGCCCGACGCAGTTGGTGATAGGCCTCTCCCCGGTTCAGCGCGCGCTGAACATAGTTTCTTAAACTGGCATCATCGATGTAGCACAGTAGATAATTCGCTTTCACCAGGCGATTGTATTCCGTCAGGGCTTCCAGCAGCGGGTGATTGCGCTTGTACTCCGAGAGCTTTCTCACCAAGGTGGCTTGCGTTGTTTTCCGCTGCTTAAGTGATACTGCAATCCGTTGGATGGTATCCCAGTGCTGCGCAATACGATGGGTATTGATTGGCTTTTTTAAGCACAGCTGAATTCGGTGTTCTTTGTCTTCCTTGACATCAAACATGTCATTGATCACTTTGCCAACCTGGGCATAGCGTGGGGCAAACTGGTATCCGAACAGATCCAGTAACGCGAAGTTCACATGGTTCACCCCATGGGTATCGGTTGAGAGCACATCCGGAATGATGTCTGACGTATTGCTCATCAACAAATCAAAGATGTAGTGCGATTCATGTTCGTTGGCGCCGATCACTCTGGCGTTGATCGCAGCGTGATTGGCGATCAAGGTCATGGCAGAAACACCTTTTTGAGTGCCAAAGTACTTCGACGAATAACGGGTTTTGAAGGTCTCGCGCCGGGCTTCGAACTTTTGACCATCGGCACTGGCGTGGATCACATCTTCCTGGATGTTGTAGTACCGGAAGATGGGTAGCTTGGCTGTCGCGTTATTGATGTTGTCGTTAGCAGCATTCAATGTTTCCAGGCGAAGATAGTTCGCCTGGATAGTGCTGAGCTGATCATAGGTACGATCAGAGATCTGTGCCATGCCGTAAATGCCTTGATTGGTTGCATTGCCGACCAGAATTGCCAACAGGTCATATTCATGGGAACGGCTTCTGGATTGGGAACCCAGCACATGAGCGAAGCAGTCAATGAAACCGGTGTCACGATCAACCATGCGCAGTACATCCGCAATCCCCGTTGTGGGAATTTGCTGGAAGAAGGGATTGTTGACCAGATGATGTTTGCTGGCCGAAGGCAGGCGCCAGAAGCGTTTACCCTGCGGATTACGCAAGATGATATTCCGGTTGTCTTCCTGTTCAAGATATTCGCCGACTTCGTACAAACGGGTATCCAATTCCATGGCCATCTGTTTGATCAGCTTTTCAGGCTCCTCCGCTAATTTTGTAAAATGGCTCTGTTGAAGCAGCGTATATTTGTTTTTTCGCCAATGTTCCCCGTCGATCAGGTCGGCGTCGAGTGCCCGGTATTTAGTGATATCAGGCAGCGTCAGCTGGCCATTCAGGCGATCAGGAATCTGTTGATAGAGGAACCACTCATAACGATCGATCAGGATATTCCCTTCACCATCCAGCAGGAATTCACGTGATTTTTTGGAAAGGAGTCTGGTGTCGGCAGTTTGCAACTGAGCGTCCTGGCCGTTGAGTTCGTTTTGTGTTTTCGCCAAGGCGGCCGCTAAGTGCTGGGTGCCGTCGCAGCCTTCGAAGCGCAGACATAGGAACAACTCTCGCAGCAAACCTTTCCGCAGACTTTCTTTCTCGTCGCAGTACTGCCACATGGCTTCATCGACCGATCGTCGCTGCTCGTTCAGGAAGAGACATACAGATTCCAGATCCCTTTTGGTCAGCAGGCTCAGTGCTTGCTGTCTTACTGTTGCAAACGGTAGTTGCAGATCAATGCTGTCATCAATGAACAGGTGCAGTACTTCAGCTGCCTTGCTGACATTTTTAGCTGCTTTTTGCCAATCCTTAAACACCGCTTCCTGCGCATAATCCTTTGCCTTCTGTTTGGTCTGTCTGACATGATGCACAAAGCCATCGGCAATACGTTCCAGCGCTTGCTGCCATCGCGTTTGAAGATAGCACAATAAATACAGCCATTGGCTGCCTACGGTTTGACGTTTCAGTTTGGCACCGTAGTAGTCGACTTTCTCTGCCAGGTGTTGCAGATTTTTCTGCGACAGTGATAGCGTGCTCAACAGCAGATCCACTTCCGGCATCCAATGCTGGATATGGCGATAGACAATCAGTTCTTTCTCCAACTCGGTTCCGGTGAAGTTACGAGCCGATTGTCGCAACTGTCGGAACGGCAGTGGACCTGTGCCGTTCACGAGCTCCGCCAGTGCTTGCTTAAGACCGCGTGACATTGCACGCTCAAGGTGCGCGGCCATGTGTTCCTGTTCGTCTCCCACCACCTGACTGATGATCTTTTGCAGCGTGCTGTAGGCAGGGATTGCGATCTTCTGCCCCGAACAATATTCATTGGCCGCATCGAAGAGGTGCCTAGGCGCTGTCCAGGCTCTGGCTTGCTGGGATAAGTAATCTCTCAACGCCGCTCCATGGTCTTTGACATTCCAGCGCTGGTAGTTGCAGAGCTGGAAAACACGTTGGTAAATCCGTTCGTTCTCTTTTGGCGTGAGATTAAAGGGTCTACAGCCTGGGCCTGGTAGAACGGTTTGATAAACGTATTTGAGGTCCTGCTTGATCTGATGAAAGCCGGGATTCAGCACCACCGGCTTGGCTTTGAAATAGCCCAGCAGTACCACCAGCATGTAACGCTGGCCCCGATGACGGAGGCTTTTAGCGATCGCCAGTTCCTTATCGTTCAGCGAGAAAAAGAAGCGTTGGTCGGCGGAGGTGAAAGCGGGGGGTCCATATAACTCATCTTGTTCTGCCTCGGACAAGATTTGGACACGTTCTTCGAATGCCATGGATTTAGCCCAAAAAGACTAAATCTTACTCAAACAGAGCCCAAATAGGGATCTCGAAAAAAGTAATACCCCTCTGGAGGCCACAGACGGCGGGGCCTCCAGAGCACTTTGTCGTTTTTGGACGGAAAATCCCTAGAACCCCTCAGTGGGACGAAATGATCCGGACCGCTGGCTCCCTGAAGCTGGGCAAAGTACAGGTTTCAGTGCTGGTCCGTTCATTGCTGAAAAGTGAACGTCCTTCCGGACTGACTCAGGCAATCATTGAAGTGGGGCGCATCAACAAAACGCTGTATCTGCTTAATTATATTGATGATGAAGATTACCGCCGGCGCATTCTGACCCAGCTTAATCGGGGAGAAAGTCGCCATGCCGTTGCCAGAGCCATCTGTCACGGTCAAAAAGGTGAGATAAGAAAACGATATACCGACGGTCAGGAAGATCAACTGGGCACACTGGGGCTGGTCACTAACGCCGTCGTGTTATGGAACACTATTTATATGCAGGCAGCCCTGGATCATCTCCGGGCGCAGGGTGAAACACTGAATGATGAAGATATCGCACGCCTCTCCCCGCTTTGCCACGGACATATCAATATGCTCGGCCATTATTCCTTCACGCTGGCAGAACTGGTGACCAAAGGACATCTGAGACCATTAAAAGAGGCGTCAGAGGCAGAAAACGTTGCTTAACGTGAGTTTTCGTTCCACTGAGCGTCAGACCCCTGTTACCGATGCAAGAACAGGGGAGTTTCATGAAATGCCTGTTTAGGAGTGATTAAAGGTAACTTGTGCGCAGAAGGTCAGGTGTGTGGTGGTTAGTACAGACTAGTGAATGTTGTAACTAAACTATATTTTTATTAAAGGCCGGAGGTGGTGTTCCTCCGGCCTTAATTACGTTTCCGTGTTCATTAGTCGATGAACTGGCGGATCAGTTCCAGAATGGCAATCAGCGCGTTTGCAAGCGCCGCTACCGCCCTCAGAACGATGATTAACAGGTTAATCATCCGTTTTCTCCATAAATGGAGTAAAGACACCGTGGCTTGCTCTTACACTGCCTGTCACCACGGCTTCTCTTCGTCTTGTCAGTTGTAGAGAGTCTCACTCCTGCCAGAGCACAGATAACCAGCACCACCTGTATTACCTGTCAGGACTTTGAACCTGTTCATGCCGCATTTAGCGGAATGCAGGGAGAGACTCACCACAGAGCTAACCCTCTGATTATACGTTTATCTAACGTTCAGGCAAACCGTTAACGCACAGTCACTGCCCGTCATATTGCATAATCAGGCTTCACGGATTATCATACTATTGTCAGTTGTAGAGAGTTTCCCTTCCGGGCTGAGTTACCAGCTCGCACCGGAACCGTAAAAAAGCCGACCTCACCAGTCGGTTTTTTTACGTCTGCGGTTTCCCGCTTCGTTTAAATCTATTTTAAATGAATAAATTGTTCTGCGGCAAACCCAACAACATAAGGACGTTAGGGTTGATGGTTGCCGAAATCGCGGTGCGTTACGTGAATTCTGAGGTTTTGCGAGGGGGATTGGGCGCGAGAATTTACGAGAGAGCGCCGGAACAATGCCGGGATCACAAACCATCACCCGTGAAAGAAATCTTCCGGCATTGCCCGGTCAGGTAGTATGACATTCATTGTCAACGGCCATCAATTTATAAGCTGCTTTGCACTTCATAAAAGATCACCACCAGGGCTGCTAAAACAGTACCGATAGCCAGCACGTTAAAAAGTACGTCCTTCATCTTTACGAACGACATACTGATTTGCGTCCTTTCCCTTTTGTCAGCCTTCTCTATCATCCAGCGTTGGTATGAATCACTGTTACTTTTTCTGTCCATGTTCCCCCCAATTCATCAATGAGCACCAGGCGGCCACCAGGCTTCTTGCCAACAAAGTAACGGCCTCGCGGCAGACGTTCGCCAGTCTTCAGCCGTTCGATCCAGTCCTGAAATGCAGGGCGCGGTTTATAGCTGTAGCGGCCAAAATCAGCTAAAGACTGCGTGACCATGACTGTTTGGCAGCCATGCTTTCTCATATCACGAAACGTATTTTCCAGTGCAGCCAGTGTATTCATTTGCACGTTCTCTCTGCTTTTCCCATGAAGATATTAACGACACTCAAAACTAACACCGTTGGCATGATCACGGCAGCATAGCCGACAGCAACGGCCTGATATATGGTCAATGAGTAGTCGGGCTGTTTATACAATACGACTGAAAAAATGCTCCCCAAACAAGCAAGCAGAGTTAAAAACAAGTAATCGACAAATCTGTTTTTATAGATAACCCATGCAACGATGCAGGGATATGACACCAGGCACAGGATAATCGCATTTGTGGTATGCACGTTTCGCTCCTTGTTATTTAAGGGTTGTCAGCCAGATCAAACAGATTGTTCAGGCTGGAACGGAATTGTTTTACCGATGATACCAGCTCCTGACTCCGCGCTTTTTCCTGCGCCAGTTCACGCCGTAGCTGCGCCATCTCGTCTCTGTTACGCTGAAGCTCAAGGATAGCCGTCATAAAGGTTTTGCTGGCTGATGCCTGTCCGGTCGCTTCCTGCGCCTTTTTAATGGCCTGATCTTCGTCGTCATTCGTTCTGATTGTGTAATGCCCCATGATACCCCTCTTTTGCTGTCATTTTTGCGCTGCCGGCGCGAACCCGGGAAGAAACTTTGCTGGCAAAAAACACTGGCCAACAGATGTGACCGGGAAAATTTGCTGGCAATTAATTTTCGATGAAAGCCAGCTCGCAATCGCCGCATTCAATATTCAGTCCGGCTTTGCCCCACACGGCAGCGCCACACCCGCGACACTGATATTTAACTTTATTTCGTTTTGGCTTGTCCTCAAATGCTGCAACTGACAAATCATTGTCATTGCTCCCGGCCATATCATCTGAATGGCTCCCGTTCTGCGGTGACACTGACGGCAGCAGAGCCATAGAAAGCACCGCTTCTATATCAATCCCGGCCTCAGCATTTTGATGCGCCAGAGCAAGCGTTTCGCGCCATTGCTCTATTACGGCGGTCATATCTTTTTGTTGTTCGACCTGGACAGGGAAGCGATCAAACCAGCTCAAAGCAAATTGCCCCTGGAACAGTTCCAGTGTGACGCGCTGGAAGCGGCCACCAGGGATCGGATAGTCATTAATCTTCTGGCCGACTTTCGCACCACCAGGTCTCCCGGTGGAGGAGGGCATAAGTCCGATACTTTCCATTTTTGCCGCCCACTGCGCATTGTGGTACGTCTTACGCGACGGGTTGCCGTAGTGGTACTGCCACATATGGCACTGCTCATGAACCAGCGTTTGCATGACTTCAATAAGCGGGTACACCGGGAAGTATTCAGGATTGAGGGCGATTTCATCAATCATCCTGCCGCTGCCGTCAGCAGCAACAAAGCGCCTGTAACTGAAATAGCCCATTGTGTTCTTGCCGCGCTGGAACGTAATCAGACAGTCAGGGAGTTCGCCGTCAAAAAGGCGCTGGTTATAGAAATCAAACGCCTGTTGTAGCTCGCTGTAAGCGCGTTCCGTGGCTCTTGTCATGCTGAAGTTCGCTTAGCGGCGTTCCGATATTCCTGTAACTCTGCAACAATAGCAGGGAACACATCAGCGCAACGCTGTGCCAGCTTAACCGCCTTTGAACAAACGCCCATACCATGTGCTCTTTTGAGCGCCTCCAACAAATCTACCAGTTCCTCGTCAGTGATTTTTTCAGTCATCTATTAAGCTTCTCCATTATCTGTATCGTACGATACAGATAATATAAACCACACCTTTGATAGCGGCAATCTTTTTTGCTGGCAATTTCACGCCCAGGGCGGGCATCGAGCGGGTAATATTGCTGGCAAAAATCAACGGCCAAACCGGCTGCAGGAAACTGGTTTGCTGGCAAGCGTGATTTACTGCTGGGCTTGACACCTTGCCATTGTATGACGCTTGTTTTAGTGTCAAATGAACAACCAGCGCGAGAAGAATATAGGAAGCAAAAGCAATGGATATCAAACCTAACTCAGTGATCCACCTGGAGCCAGGCGAAACATTACCCGGCTATACCAATCTGAAACCCGTACCTGATAAGTATTTCGATGAGTTAAAGGCACTCATAAATAAACTCAATACTCAATCCGGTAATATTTACCTCAAGTTAAAACAGATGTACGCCTTTTTGGATCGTTTTAACAAAGAATTTGTGAGCACGTTTACCTCCTGTCAAAAGGGGTGCTCTTCGTGCTGTAAAATGGATGTTCATTTAACCGCGCTTGAGGCAACACATATTGCACAAGCAAGTAAACTTACTGCCAGAGACAACCCGCTTACGACCGGGCATGAGTCAAAATGTCCGTTTTTGTCTGAGAAGGGAACATGCTCAATCTACAATTACCGCCCGCTGTTATGCAGGACATATCATGTACTCACTCCGCCGGAGATGTGTAATGACCTGGATGCACAAGTCATGCAGTATGGTTCTCAAAGCGCGAACATGGGAAATCACATCTACAAAACAATAGCAGAGTGGATCTATTTTCAGACGTATCACTGCACTGGTAAGTTAGAAACCAAAGATATCAGAGACTACTTCCCTTATCCCCGCGAAGATATCCAAAGATTTCTTCACCATAACCCCCCGCGCCCTTTTTGCTAAAAAAAAGCCTCTGACGCAGTGTCAGAGGCTTGTTAGATTACTGTTCGTCACCAGCAGTGCTGGAAGCATCTTTTTTGATGAGAAACTCATCAAGAGAACGGCCAGCTTTTAACGCTTCGTCGATTGGTTTTGGTGTCCGGCCACGACCAGACCAGTATTTCGTTTCGCCGTTTTCATCAAACTGATATTTAGGCGGCGCTTTTGGCAACGTCTTTTTGCGTGTTTTTTGCGCATCTTCGGACAGGCCAAGCAGTTCTTCCGGTGAAAAACCTTCTCCGGCAATCAACTGGAGCAGTTCCTGGCGCTTTCTTTCGCGCTCTGCGCGTTCTGCTGCCTCACGCTCTGCTTCTTCACGACGTTCTTCGATCACGGCGTTAAGCTTTTCCTGCATTTCCAGCAGTTGTTCAAAGCTGGTTTCTCGCGCAAAAACACGAACAGAGCGAATATTCAGGAGTAGCTTTTTTGTTGCGTCAAACGTTTCATTTTCACTCATCGAGTTAATCCTTATTTTTTACTGAACTGAAACCCAAGTTTACCCGTAGTTTTATCCTGCAAAACAATTGCAGCGTCAAACTTTTTCCCGGTTTTATTACTGGTAAAGCCTTTAATCTCTCCGGTTTTACCTTTTTTGATTAATGTCTCAACCTGATTTTGTGTCAGTTTTTTTCCTGACACTTCAGACCAGATTTTAAACTCGCACCCGGAACAGAAAAAGCCTTTAGGCCGGATAATAATTTCTTTACTGCATGAAGGGCAGGGGGCTTCGAGCCGGGACATGGAACCTTCTGATCTGGATTTTGCTTCCATATCCGGTTTGCCGTCTTTGTCCGGGAATGTTGTCTTGCATTCAGGGTAGCAGGAGCATCCCCAGAAAAAGCCATTTTGCCCCTTACGCTTACGCAGGAAACCGTTGTTACAAACCGGGCAGGGCGTGGCATTTGAAGAAATCGAAATTCCGTTCCTGTCCAGCTCGTCGATTAACCCCTGGACGTACTCATCATTTTCCTTAATAAACTCCTCAACTGTCAGCTCTCCGGCTTTGATCTGAGACTGCTTTTCAGCCCACAGAGCGGAGATATCCGGTTTAGTGATTTCAGGAGGCAGTGCAGCGCAAAACTCCTGGCCTTGTTTTGTGGTTTTCCATACCAGCTCCGAATACCCCTTTTCTTTTTCGATGGAGATAAGCCCGGTATTTGCCGCCAGTTTCTCAAGAATACCCGCGCGGGTGGCTTCCGTTCCGATACTGCCACGATCACTGCTGCCCTCGTCTTTCGCTTCCAGCGCCTTTCTGAGAGCCGGATCATCAATGAATTTTGCCGCACTCGTCATGGCGGCCAGTAACGTTGACTCTGTGAAATAGCGAGGTGGTGTAGTCTTTTTCTTGTCCACGTCTGCCGATTCACACAGGCCGCTGTCATTAAATTTCAGCAATGAAAGGTCAAAACCGTCCGTTCCGGCATCTTCGGTTTCGTCCTCGTCATCACTATCCTTACCCAGCGCTTCCCAGCCCTTTTGTACCAGAACGCTCTGTGTGGCCGTAAAGGTATCCCCCTTAATATCAAAGTGGATTTTCGTCTTGTTGCGGATCGCATCCGGGTAGAACAGCCCGATAAAGTAGACGGACACCAGGTTATAAACGTTCCGCTCTTTTTCATTAAGCTGAATACCGGCTCCGCTTTTTGTCGTCGGGATGATGGCGTGGTGCGCCTCAATCTTACTGGCATTAAAGGCTTTGTGTTTCTGGCCTTTATCCATATCTACGATCGCAGACGCCAGTTCGGGTACGGTGGCCGCTATAGCAGTGGCAATATCTCCGGCCTGGTAATAATGCTCATCTGAAAGATAGCGATTGTCAGAACGTGGATAGGTCAGAAGCTTGTGAGTTTCATACAGCCCCTGCATCACATCGAGCGTATCTTTTGCAGAATAACCAAATCGCTTTGCGCAGAGCTGCTGAAGCGTGGAAAGGTTAAGCGGAAGCGGAGGCTTGCTATTTTCCGGCTTTGTCGTCGCGATAGTAACAATAGCATCCTTACCCGTAACACGTCCGGCAATATGCGCGGCCTGAGCTTCGGAGATCAGGCGTTTTTTCTCGTCTATCTGATCGTCGTCTGTTGTCTGATATTTCGCCTTAATCTGATGCCCGTTCATCGACAGACTGGCGAATACATCGTAATAAAAGCTTTCTGTGTGATTCTGGTTCGCCAGCGTGCGCATGTTCACCAGACCAAGAACGGCACTTTGTACCCGGCCAACGTTCAGAACGCCGTCATAGCCCTTCTCACGGCCTTTTAATGTACATCCCCGCGTCATGTTATAGCCAAAACCCTGATCGCACAGGGAACGCGCCAGGGCGCTTAACGTCATACCGCGAAATTTATCGTTAGGTTGTAAATTCGACAGTGATTTTTGCACTGGCGCAAGGTTGAGGTCGGCAACAAGCAGTCGCTGGACGGGTTTCTTGTTTCCGGCATAACCCAGAATCTCATCAACCAGCAAACATCCTTCATCATCGGGGTCGCCTGAATGAACGATGCTGTCGGCTTTATCGATAAGAGACAAAATAATGCGGGTCTGTGCTTCTGATTCAGGCTTGATTTTATATTTTGGCGGATAAACCGCTTTAATGGGTAAATCACTATAACGCCAGTTGGAATATTTGTCGTCATAATCCTGTGGGTCGTAAAGCTCCAGCATATGACCATAACACCAGGTAACGACATCAGAACCGTGTTCAAAATAGCCGTTTTTCTTTTCTGTATTGGGATTTCCGCCCAGTCCTTCAAAAATAGCCTTTGCTAAAGACGGTTTTTCTGCAATAAAAAGTCTCATTAATCAATCCTCAAGGTAACTTGAATAGTCAGGCTGCGCACCATTCTTTTTAAAGATTTCTTCCCTTTCCTCTATGTCTTCTTCTGCCTCCTGCACACTAAGGTCAAGGGTTCCTTTCAAAAAGGCACGCGCATTCTTGATTACATCTACTGTTAATTCGTTTTTTGTTCCGCGAATCATGCCAATAGCAATATTTATTATCTCCTTTATTTCAGCAGGAGTCGTTCTGTCCAGCACCGCGCCAAGTTCTTTAAGGTCAATATTTATATCGTCTGGTAAGTACATTTTGGCACAAGCGGCAGCGGCTTTAGTCCGAAAAGCACCTACAGATATGATTCTGTCAATTCTGCCAGGGCGTTTTCTGATGCGCGGATCAATCCGGTTAGGATAGTTCGTTGAAAAAATAACATATGTCCCGGCAAGATTTCGCGGTGTATCAGCCCCATCAAGAAAACTTAACGTTCCGGCATCAGCCCGATAAAGTTCATCAACCTCTTCAGCTATTATGATAACGGGTATTTTCTTTTGAGCTGCGGCATAGCAAACATCTTTAAAGTAATCATCAGCATAAACAAATACATATTTATCCTGATATTTTGCCCCCAAAGCTTTTGCGATAGTCGTTTTCCCTGTGCCCGGAGGACCAGTAAAAAGCACTTTTCGCATTCCTGACTGACCATAACGGGTGTAGAAATCAATATCCGAAAAGAACTGATTAAAGTCATTCTCCAGCTCATTAAACTTTGGATGAACAGAAAAACGTAAAGCGTTTTCTATTTCTTCCTTCCTTGCCTTACCGTGATAATAAACGCCGTCGTAACACCCGATTTTCCAGATACCTTTACGGGGTAAAGAGCGGTTTAATTTAGCGCGTCCAGCTTCGCAAAGATTATGAAGCATCGACATACTTTTGGTTGTGCCAACTGCGTACTCCATTCTGACTTTAGAGTTAAGAATGAAGCGAATAATATGAACCACTTCATTTTGACCAAGATGAATCCGGTATGCTCCCGAACCATCTTTAAACATAAGCTTTTTAATCCTCCCGGCTTTAGCCATAACAGGGCTACTTCCCGTATGAACGACGGTTGAAGCAACGGGTTCGATATCAAGATTCGAATAAATTTTACGATTCTTTCTGTTTATAAAAATCTCTGCCACTTTAGGATAGTAATCACAATCCTTTACGACAGTACCAAAATTATCTTCACCGTAATATTCTTTATGGAGCCGTAAAGCCTGCTCTACCAGAGTTAATTTACCCATATGCGCACCATGTATATTAAAAACCGTGACGTTGCCTGAAGATTTTCTTCAGGCAGAGGAGGGGTTGGACAAGCGAAGCGCGTCAGTTTTGTTTATGACGCCACTCCCACGGAGGAACGGGATTACTGTCAGCCCACAGGCCGCGCTTCTGTGTCTGAGCTTCCCGTTGCAGAGCCGGTAATGAGTTATCGGTGTTGTACCTGTCATAAACCCATGCAGCCCCTTTTAGCACTTGCTGGCGGTTAGCCTCAGTGCCGTTCGCTGTCACCACTCGTCCCAGGACTCGCCCGTAGCGATCTGTTTGCGTATAAGAGACGGTAACTGATTGCCCTGCCAGCAGGGCTTTAAGTTGATTGGTTGACCAGCGACCAAAGGGTTGTTTTTTTTCCGGTGCATCGATATTTATCAATCGGACTCGAATCGGAACTTCATACACAACAATTTTCGCGGGTAGTGTTTTAACTTCGATGGTATCTCCATCGAGAACGCGAATAACTTTCCCCTGAATCTGAGCAGCACACAATGTAGCTGGCAACAGGGCAACAGCAAAGGAAAGAACATTAAAAAAATGTCTCATTGCTTTCTTCCCTCCCATATTGCTGAGAGTCTTTTAAAAATGGATAAAACGACGACTAAAGCCACTGCGATAACAGCCAACGAAGGAAGTACATAGCTGTATAACTCAACCATGAAATTAATAAACTCAATGAGCGATTGCGGCTTTAAGGAAGCATCCCACAAAACGATAACAAAACGATAAAGCCCATAGACAATTGCCGGAAACCAGACCATAAACAACAAAATTTCAGTAAAAACAGAAGCGACCGATTTGACACTATCGATAATTTCAATTCCGGCAATTCTCACACTTCTTGCATCAGGTACGTTAATAATTTTCATCCTCCCACCCCTGTAAAAAATGTTCGCAAGTTCCAGTCTCAAACCTGAAACTTGCGTATTCACATCAGAATTAAATCAAACAGAATTAAAAGTTTTTCAATCGGCCAAAGCTTGAAATAATTTTACTTACTGTACCTGCATCAGCCGCAGGACATTCATTTAGTTTTTTCTTTCTGGCATCTGCCGTGTGATCGGGTAAAAAACCATGTTTATTCTTTTTCCTGATTGACATAAAACTTTTATACATCGGGTCACATTCTGCCGGACTACTTCCGTCAAGTTTTCCGGCAAGGCATAACACCATTGCACATGGATCGTTCGGGTCGGCCTTTTCCTCAGCATGAGCAGTTCCGGCAGCGAACGCACAAAGCAGCACTGCCGTGGTTACGGTCTTTTTCATATACACCTCATGGATAACGGGCAACTAACGCCCTGCGGTTGTTCAACCCCGGCTCAATAAAATTACAAGAATTTTATTAATCCGGCGTTATCGTGATAAATAAACACTGGTAGTCCGGCAAGAAAAACAGTTCATTTTATAACGATACTGTATGACACCGTTTGCACCGTTCGCCCTCAACCAGTTTTTAGCTGTGAAAAAGTCTGTTCCGTTACTGGTTTCAACAATCACTGAATCAGAATGTGCAACAGAATAAAAAACAGCATCATCGTAAAGGCGGTTTTCAGGCCTGAAATTATGGATAACCTTAACCCACGATGAATTAACCTTGTCTGATTTAATAACTGAGTATGTCGGTTGCCAGTCCATTAATTGTGTATTCACCGTTTCAGGATTCTGTTCCCAGTCAACCTGTGGTGGTTCAGGCGGAGAGGAACAGGCAGAGAGAAAAAGGACACTGACCATTAACATTTTTTTCATAGTAAAACCTCTGACTCCAGCTCACCGACGGAGAGCATTTTTTCTTTAGAAGGATATTTAAGCCCTTTAACACCCAATACTTTTTTAGTCTTATTCAGAGAGGCGGTTAACTCGGTAAGTTTGGGGCTTACCATCATCAGCCTGTCCATAAAATACGGGTCGAGGAAGTAGAGGGCTTTCTCAGCTTTAACCGGGTTTTCACCTTTAAGAATGATAAATTCTTCTTTAAAATCCAGCGTTCCCAGCTCCTGCGGGAGCACAAGGGCGCGCTGCGCCTCACTTTCTGAACTGCTTTTCGACGTTGAGCGTCCGCTTGTCTTGCTTGAACCTGTCGATTTGGTGGTGATATAGCCCAGCTTTTCCGAAATCTTTTTAGCGTCATCTTCTTCACTGACAGCGTAGATAATCCGGCAGGGATGCGCACTCATCAGCGTTTTGGCACCTTCAACACCATAGATTTCATTAAGCTGACTGATATTCTGGTAAATCGTCAGAAGTTTGAGTTTAAACCCGGCAATATAACCCGAACCTTTTTTAATGATTGGCATGTAACCAATAGACGGAAACTCATCCAGGAACATCAGACAGTCATGTTTCAGTGTCGGGTCAAAGTCAGGGTTTTCTCGTAACGTGACTTCCACAACGAAGTTAAAGAACAGGTTGAGGAAGTCATAGGCCAGGGACATATCTTCAGCATTAACGCCAACATAAACAGTGATATCTTCCCGGCGCAACTGGCGAAGGTCGAAATCATTGCCGTCTGTACATTTGCGAACAGTCGGAAGATAAAACAGACTCATTTTCTTACGAAACGAACCATCAATACTCGAACGTTGTTCGTCTTCGGTTTCGTGATACTCCCTGATTTTGGTCAGGGCATCGCGCAGATGATACAGCGCGTTTTCATCAAGTCCTTTTGTTCCCTCCAGGTCTTCTCGCTTACTGAGTATCAGCTCCCGGTCGATGTTGCTGTACAAATCGACTACAGAACCAATTGAGAAAACAGGTTTAAGCCCGAACTCACCAAGCCAGGAGGGGTCGTAGTTAATAAAGAAATGAAGCAATTTTGCCAGCCCCGTCCAGTACTGCCCCGCGAGGTTATTAAAGTGTGCTTCCGCTCCGGTCAGACCGTAAGACGGGAACAGGATCTCAATCAGCTTCAGCAGGTCTTTAGCCCCGCTCTCTTCTTTTAAATCAATATAGAAAAGCGGGTTAAACTGATGCGTTTTACTGTTGAAAGGGTCAAGAAGATAGACCTTATTACCCAGCAGTTTTTCACGTACCTTACTGGTGATTTTCCACAGTTCCTGTTTTGGATCTAATGCAATCAGGGAATGTTTCCTGACCAGCAGGTTTGGAATACCTATTGCAGCCCCTTTACCTGCGCGGGTTCCTGCGCCAAGTGATACAAAATCTGGTGCGGTATACCACAGGTATTTACCTTTATATGCACCTACAAGAATATCAGTATCGTTTTCTTTCTCCCATTTCAGAAGTTTCGATTTTCTTAAATCATTATCACTGGCAAACTTCGCGTCACCATAAAGAGCAACATCCGTTTTATTCAGTTGCCAGATAATAAAAACAGGCACAATGAGAGACGAAACCATACCTGATAAAAGAGAGGGGATTGCGGTTAACCTGATATCTGTCCGTATATTACTCTCTGTTATTATCCGCCACAGCAGCATGGAATCAAAATTCTTCCATATATAAAGCGGCGTTTTTCCGTTCAGGAAGTAAACAGCAACTGAGCCAGTATAATACGTCACAAGACACATAACCAGACCGATAAAAGCCCACTGGCCTTTATCGGGTAGTTTCAGAGACATAATCACTCCTTCAGCTTTTCAAAGTATTCCTGAAAATGAATATCACGATAGTAAATACCCGATGCAAATCGGTTATCTTCCTCATCGATGTACTTAATACTCATAATCACATCGACGTTGCTTAACACCCTCCGCAATATAATGTTATAAGGCAAATTCTGACATTCAGGGTTCATATAGCACCGTTGAACCAGTCCAAGCACAGCATCTTCAGGGGTGCTCTCATGGACGGTTGTAAGGTTCCCTGCATGGCCGGAACTTGAGCCTTTGAGAAAATCCCAGGCTTCCGCGCCGCGAACCTCAGTCATGAGGATTCTGTCCGGGTTCATACGAAAACCAGAACGTAAAAGAGAAGCGGAACTGACAGTGCTACTTTCCCCTTCTGACGGGTAATACAATTTCACATGATTTTTGTGGAACCTGAACTTTGCTTCATCTGTATCTTCAATTGAAATACATCGTAAATGATGGGGTATGTACTGAAGACAGGCATTTGCAAAGGTGGTTTTACCCGCCCCCGTTCCGGCACAAAATACCATCGTTTTACCCTTAACCAGGCATTCAGGGACAAATTGTTCAAAACAATTATCTTTAAACATTGAAGATAATTCATCTTCTTTATCCCTGAATTTCCCCCCTTGATTTAGCCTTGAGTAAAACCCTTGTTTAACAAAGGTATCATGGTCGATAAACACACTGGACGGCTTACGGATTGTAATAGACACCGTTTCCCGTTCCGTTGCCGGGGGAATAACTATTTGTACACGCTCCCCTCCGGGGAGCGTAACAGAACGTAAGGGATATTCTGGCGTAACCGACCCACCATCATGATAATCAGAAATTGCCCCTGCAAATGACATACAATCTTTAAAATCCATTTTTAATTCATGCTGCTGCCATTTCCCACTCACTTTAACAAACAGTTCATCAGGCCGATTTACCGCGATTTCAGTTAAACCATCGACTTCATTAAGCCAGTGGTGGAAGTATTCATCTATTATTTCATAAACAATATGTCTGTTCTCTGCATCCTTCATAATTCACCTTTTCCTTTAAGATCTTCAATAAAACGCATGACTTCTTCGTAATTAAATCGATGCGTTTTGATTTGTTTCAGTGGTTTGGGGAAAGGAACGCCATTTATTGGTATGCCCTTACGAATATGATGGTATATTTCGCTTTCCCTTATTCCTAACAGCATTGAAAACTCTTTGATATCCAGCGTTATCAAATTACCTCCGGGTACCCACCATTTTTAATTTGTAGATGCTGGAGAAATCCAAATCCTGACCAACGATCAGAGTAATGATTTCCCCCTGGTTTTTATAAAGCGTTGGAGGGATATTTACGCTGTTTGCAAACGCTTCGCGTGCTATATCAGCAAAAGCCTGGCGACTGTTAGCTGTATAGTCGGTCTGATTGTCTTTATTACTTTTAGCCGCTCCACTTGCGGCCTGTGCCACATCGGGGATCATGCCAACCATCATCGCGCCCATAAAGCGATCTGCAAAATGTGTGTCAATCCAGCCTGAAGCCCCGGCCTCACCCAGCGCTCCCGCCGCCTGTGTATCAATCAGCGGAATATCAATGAAAGGTTCCTTACGTGTTCTAAGCTTCGTAGCAGCGATAAACACGGCTCCCTGACCATGATTGAACGTGCCCGTTTTATACATCAGATATGCGGCTGTTCCCTTTTCAATTAGCTTTACATTCCTGTTTGCACTGTATACGTCCTCATTAATCGTACAGACCAGCTTGCCAGCCAGATCAGAAATAAATCGTCTGTCCAGCGAGCACTTAATCGCGGTATTTTCAGGAATGAAAAGGTTAGGGTCATAAGGAACGCGCGTAATTTTAGAAAGCTGCGCGGGTTGCCCTGCATTAACAGGCTGAACCTCTGCCTGTTTGTCACTGCCGTCCCCCTCGTTCCTGGTATTGCTGGCCGTTGATGAGCTACCACCGGAATTTGAGGAAGCAGAGCTGCTCCCCCCATAGGCCACATCCAGCGCACGACTGAAGTTTTGTTGTACTTTTTCCGGTGGCGTTTCCGCTTTGCCGCTGCTTTCCTTCCGTTCTTCCGTCTTAGCCGGTTCTTCCCTGGTGTTGAATGGATCAACATCATCGCTGAGTCCTGTCGCGCGTTTTACTGAACCAGTAGATACCGCATTACTGGCAGGCTTATCATCACCGCTGGACATTGCACGAATAATCGACGGGATATAGTAGACAGACACCCCGGCGGCCAGCAGAAGGAAAGCGGCCACAATAATCACCAGCCCCTTACGCGATTTTTTAAGCTTCTCGATACCCAGGCGTTTTACTGATTTACCGTCATCTTCATCTCCCTTATCCGTTTTATCGTTTTGCTCAATTTCTTTCTGATGCCGTTCCCGTAGTGCCTGTTCAATTTCTACAGCGGTCGGGATTTTATTTTCCTGTTCGCTCACTGGATCACCTCTTTCTCAACGTTCGGGGAAACGGTATCTCCTGCCGGAAGACGGACTTTGCCAAAGCCCCGGTTTTCAACCCCAACAACCTGATTTCCCATACGCAAAACAAAGCGCTGGTCTACAGGAACACCAACAACGGTGAAACTACCGCTGTTTTTGACTACCGGGTTAGTCAGCGTTTCCTGTTCGCCGCTCATAACAAAGACGCTGGGGATTTTCTTCAGGGGGCTGAAGCCAAACCAGGTATAGCGGCCATCGTCATACGCATAATCCGGGCTGATTGACTGGCTGCCGTCAGCTACCCGCTTCCAGTAATCCCAGTTCTTGGGCGACTTCGCATTTTCAAGAGAACGGTTGATTGCCTGCTCCTCCTGCCGCTTGTTAAGAGCCTCAATCCGTTTCTTCTCGATTTCGGCTTGCTCCTTCCGGCGTTCCTGCGGGTACTGGTAATTAACAACAAACGCGATCTTATCCGGCTGCCGGAACGTTCGGGCGTTCAGCTCCATGCTGTAATTACGTTTTGTTGTGCGAACAAACAGGTTTGTTCGCCAGCGTTCCAGGTCGTTTTCAGGATCAAGGGCTACACTGACCACCTCTGAAGACTGGTTGCCATTGTCATCGGTACTGTTCTTCTGTACGGTCTGTTTGACGGGCTTCACTTCGAGATACACCAGGTTATCTTCTTTGTTCACTTCCCAGCCCTGGGGAAAGCCGGTTCTGGCACTGATGACCGTTTCATCCTCGTCAAATACCAGTGTGGTTGTATAGCCAACCGCACTGTTTATGACTGTCGTGTTTTGCGGGTTATAACTGACAATCTGATTACGGGGATCAAAACGGCTGGCTGCTGGCGTAACGGCTGCATGGCAGACGTGCGACACCAACAGAAGCGCCACAATTAGCGCTCCTTTATTCATTTTCATAGTTTTCACCCTTTCACTTCTTTATCGCGTACATAGCTCGTTACAACGAATTTAAGCGGGTTATTGTTTCGGTCACCCGATGCCATTTCGACCTGTGGTTCAATACGGTAGGTCAACCGGACGTTCCAGTATTCATAATCATCCTGGCCTGTCGCGACATTTCGAATGGTTAATTTAAACCGGATATATGCTCCCATATCCTTATCATCGCGCCGGGATGACGGACTGATAATCACGGACGGATTATCCTGAACAGATGCGGTTTTTTCCGCTTTATTATAAATAACCTTTGGTGACTGGTTGCCGTTGATTAAAGCGTTGTAATCCGTCGCCACATTCTCAGCGCTGTATAACAGAACATAATCATAATCATGCTGGAGACTGAAATAGTTATACCCTTCACGACGCTTCACATACTGAGTAATGAAGTATTTCGCCAGCGCTTCATTTTCAGAGAGGTTTTCTTTTTTAACAGAAGTCAGTCTTTCAGCAACGCCAGTGACACCATTAATAAGATAAGCTTCGACAACCGTACTCTTTAACGGTAGCGCTCCATTCAGTGCCAGCAGAGACAACACACCTACGGCAATTCCTGCAATACCACCGAATTTATAAATGCGGTTCATTTTCTCCTGCATCAATACATTTTTCTCTTCAAAAGTAGTTGAAGAGTCTGTTACTTCTTTTTTCTTGTTAAAACCGGGAAGTTTTAATCCTGAGAGTTTAAACCCCATTGCGCATTACCTCTGCTGAATTAACAGGTTCGCTTTTTCCGGATACCGGAGGTTGTTTATGTGACGCCTGGCATCCGACAAGGCAAAGCGTCACGATAGCCGTGATAAGTAGTTTCATAATGTGAGAACCTAAATAGAGGAGTGAATATTTACTGCGGAATACTCAGAGTTGACAGATTTAAATAATAAATCGGTTAACCTCCATATTTCTTTTTCGCCGCATCTATCGCAGCCTGGCGAAGCCTCTTAGCGCCAATATTAACGCCCTGCCCGGTCAGATTCCCCAACTTACCGGATACACCAGGAGACTGGTTGAACCCTCCATCCTGACGGCGAACGCCTTTCCATGCGCCAATCCCTGCATTTTTACCCATACCAAGCAGATTACGCCCCATACGGGAAGCGCCGAACACGCCAGCACCAATCCCCATTGCGGCTGCCCCTTGCATCGCGCCTTCAACACCTACCCCTGCGAGCTGGGAAGCATAGGTTTTGGCTTGCCAGATAATCCAGGCCATAAATGCACCTGCAACCCCGGCCTGAGCACCTGTTGATATCAGATTTAATTCATCTGCGTTAGCTATCGAGATTGTCAGAATGCCATTAAGGAACGTCATTCCCGCTTTAATTGCCAGTCCACAAAACAAGAATATGAAGCATGAGCTAAATATTAACTGAAGCCAGCTATTAAACATCTGCCTTAAAAAACCAAATGAAAGACAAATAATAAACAACGGCGCGGTAATAGTAAGTATCTTAATAGTAACTTCGGCGGCGAGATAAACTATTGAAGTTAGCAATAATGCAATTATCCCTCCAGCATATGTTAATAAAGATGCGATTGCCCCATCTGTTTTCACATACTTCGAAGTATCTTTGGACATAAGATATGCAGCTACCTGCTGAACTTTCTCCCAAAGCTCGTCAACCCATTTCCAAGGATCACCTCCTGCAAATGTTTCCTTCAGCCCATCAATGGCCTGAGTTGCACTATCTAACCACCCCCCCATATTTTTGACAAAAACAAGAATTAACCAGAATCGACATAGATTCCATAAAAAATCCTGCACTGCGGTTTTTTGCTTCCCAACTATTGTGGTATAAGCATACCACAATATATAAATGGAAATACCATACTTACCCAACTGAGACGCTATTGAAGAAAAGTTATCTAACTGACCAGTCAAAGACTGGTCAAGAGTTTTAACAATAACGTCATGAGCGGCCTGGAAATACCTAGAAGTTGCCATAGTGGCTCCTTTTAACTTCCATCTTTACCCAATACATTTTTGAATGCATCTTTATAGTCAACATGCTTAGGATGTAATTGCTTATACCTAAACAAACCTTCCTTAGCGTTTTTACAATTTTCGCTATCACTCCCAGACTTTTTGCATTCAGCTACTTTTTTCGTTGCATCGTCTACGTGAGACTGCCACCAGTCTACAGATTTAACCTCTTCACACCCGGTAAGAAAGAATGTAAGCCCCAACGCCGCAACTAATCCATATTTTTTCAAAATGCCCCCTCAGTTCATTTCATAACTCTGCTGCTCAAATGCTGCCTTATAATCAACAACTGGAGTTTCTTTATTAACAGCCCCTAATTGTTTCTCCTGGAAAGCAGCCTGTAACATTTTCTCCTTGTATTCTGCAAGATCGCGCTGCTTATCGCGATACATTTCATACTGAAATTTGAGCTTGTTAAATTTCAGGCTTGCCAGAGATACAGCGTTTGTCGCGTCCTGCGTGGCTTTCGTGTCCTGGGCGTTCTTCACCTGGTCGATAAGGTCGGCCATTTCCTGATTATCTTCACGCAGTTTACTTTCCAGCTTATTGCCGTACTCGATCCCCGCCAGTTGGGACAGGAACGAGGCTTCACAGCCCCGCACCAGGTCGCCCGTATATCCCAGCCCCTTACAGGTGTTTGTCACCTTATAATCGGCCAGTAGTGATCTGGCTTGCTCAGACAGGGTGGCTTCCGGGTTCTTAATGTAATCATCAATGAAGGAGTTCCCCTGGTCGTAAATATTCTCCAGCTCCTTACTGACAGATTGCGCAGACTGCACCAGTCCCTGAACATCACGAATGCCCGTTTTGGTCAGCAGCTCCTGCTTATAGGCATTAATCTGTTTCTGGTAGTGGTTCACGGTGTCCTGCCACTGTTTGAGGCGTTCTGTCCAGCGCTGCGCTTCGACAGCCCATTCAGGGTTTGCATCAATGGCAACGGGAACACCAGCCTGAGCACCAGAAATAACCGTAACAGAGGCGATAAGTGCCGCAACGATATGCTTTTTCATAGGGATTCCTTAAATCAGGTTTGCACGTTCCAGATAGGTATCAAGCCACTCTTCCGGCTTCATACCCTCTTTCCAGATTTCATCGAACAGTTCAAGCTGGGGTTTCGATGCGCTGAGAACCGGTAGGTAATACGCCGCTTTTCCCAAATCCAGTCTTGCGAAAGCCGCAAAATCATCACTTTCCCCCTTCCTCTGTGGGTTTTTGACGACCAGGAACTGACGAGATAACGGGTCAATGGCTTTAATTTTGTCAAAATAAAGTTCTCTCACTTCCAACTCGTCTACGTACTCACTACGTTTCGCTTTGGGGTTCGCCAGATAAATATGTGTTGCGCACTGCTCACGAACGGCAGCCGCGATAGGTGATTTAATTAGTTCATCCGGCGATTGTGTCGCTACGACAAGCACCATATCCAGCTTACGCGCGGTTTTGAGTTTGTTATAAACAAAATCTCTGAACGCTTCGTTGTTAATCCATTGCCAGAACTCATCCATGTAGATAAGGAGTCGGCGGCCATCAGCCAGCATGGTGACACGGTAGATGAGATAGAATGAAGCGGCGCTGGAAAGTACTTTGTTATCCAGAAACTCCGTTCCGTCTATACCGAAAACGTCCAGGTTGTCCACATCAAACGTATCGTCCGGGTTATCAAACACCCAGCCAAATTCCCCTCCCTGCACCCAGGCATTCAGGCGTGCTTTAAGCCCATGCCGCCGGGTTTCAGTATCATCAGGCTCCAGAATGAGTGGTCGCAACTTACTGATCGGATAAGAGCGGTTTGACCTCTGCATCAGGCGCTCAACGGCATCAGAAATCAGGCGGTTCTGGTAATCATCAAGCGGCTCACTGTTAAGCGTACAAAGTAGCCTCACCAGGTCTTTCATAAAGGAAATATTTCGTTTGGTTGGTGGCAAAGCCGCAGGGTTCCAGCCAGTCGGCTCTCCTTCTTTCACCCGAAAGTAAGCACCTCCCATTGAACGTATACCGGGTTCCGCTGCCCGATCTTTGTCGAAAAATACTGTAGTTAACTTCTTTAATTTCCTCTCAGGTGGAAAAGACTCCGGCGTTCCGAACTGCTGGGCGGCGAAAGCCTTAGTCATCATCACTACCGTTTTACCCACGTTTGACGTACCAAGAATTTCGGTATGCCCCAGCGTGGGGTTTTTACCGAAGTAATTTTGATGCTCGGTAGTCATGTGATAGTTCAGGTTATAAATATCATTTCCTGAGCCGCCCAGGGTGATAAGGTTGTTACCCCAGGTATTTCCTTTCTCCTTGCCCGTAAAGAAATTATGGAAGCTTTCCATTTCGGCAAAGTTCAGACTGCTGATAGAACTCAGGCGTGGCCGTAACGTGTAGTTACCAGGTAACTGAGCAAAATACGCTGCGCCAAGACTCAAGGTTGAATAGGTTACGATCAGCCCTAAATCCTCCAGGGTCGTCGTTACAATATTGGTGTCCTTCACCAGACGCTCCGGCGAATCTGCGTAAACAATCAGTGTCTGGTGACTTTTCCCGAAAGAAATATAGCCGCTGGAGACCATATCGAGACCAACTTTCAAATCAGCGAGCAAGGATTTAGCGGCATCATCGGTCATTTCGAGTTTGTCGATCTGATCATCCAGCGCCTTGATTGCAGACTGCTTGTCTATCGGAGTTAAAGAGGAAGTCTGTACATACTCAACCGGGAGATACATCAGTGCATCAAAAATTCCGGCATCCGTTTCCTGAAAGTAGTCCTTGATCTCGATGCAACGAAAGTACCGGGCATGGTCAGAGGCAGTGATTTGTCCAGCATCGTTACCAAAGAACAGGTCTTTACCACCCAGGTATGTATAAAACGGACTATTGGTGACTCTGACTTTCTGCCAGCGCCCGGATAGCAGTTTCTGGAACAGCGTAAGCTGTTCTGAATAAACTATATTATTTTCTTCATATAACCCAAGACGACGGGAATGAAACCGGGAGAGATAGGTGCTCAATCTGCCACATATTTCATTCATATCATTAATGGGTTCTTCGAAGATATTTTTATTACCCTTTTTCCTTGAAAGAAAATGCGTCACTTTATCTTCAGCGCTGAACGGTTTATAGCATACGGTTAAGTATAATTTGTTTTCAAAATACTCAGCCGCCGAAAGCGATTCGTAATAATCGTTCATCACTCTGTTGACGAATGGGATCTTGCTGTCAAATACAGGTCGCACTTCTTTTCTAACCCGAATACGATGCGTATAAAAGGTAACGGGTTTCCCATCAAAACTACGTATCAGCGTATTGAGCTGGTCAGTAAGCAAGGCTAGATCCGCTTCATCTACACATTCAAAATAAGCTCCGTCAATCTGCCAGGTCGCCAGTAGATCATGGTTTCTGGTAACAATGAGGTTATCAGTGATGTGTGATGAATACGGGATTAACTCTTCAAGAGAAGACTGGTCTTTAAGCTTCATAAAATTGTCCACTTTCGATATATCGACTGCGTCGTACGAACTCCCCGCATAATGGACGGCATTGAAGCGCTTGTTTGCCAGAGGATTACCTTTGATTTGCATTCGCAGATAAAGCAGGTCAAAAAAGCGTTCATCTATCTTCGTAACTCGCTTGATGGCATACCAGCCAGGGAGGATGAGGGCATACATAGCCTCATGAATCCAGACACCAAGCAAAACGCAGATAACGCACATTCCAAGAAAGGGGTAGAGCGGAACGCCCAGCCCCCTAATCAAAGCGGGGCGTGTCAGCCCTTTAAAAACGGTACTCATTTTTACGCCCCCTTTTATTACATATTACGCTAACCAGTCAGGCAGATATGGCGCAACAATAAAGATGATAATCCCGATAATTAAAGGCGTAATCCACGCTGCATGCTGGCGTGAGAAGATAGCGATATAACCAATTACCAGCGCAATGATGGTGATTGCTGGCTTACGGATATCCGTCAGAAAATCGATAATCTTCTGCAAGAAGCCAGTTGTTTTAGTGGATACATCATCAGCAAAGGCGGGTTCTGCTACACAAAGCATCAATGCAGCCATAAATACAGAAAGAGTTAAATAACGTTTATTGAGTTTTAACATCGTTTGTTTCCTTTTCATCTAAAACAGTGCCACGCATGGCATATTGGGGATATACGGTAACTTCTTTAGGTTTGATACTCTGTGTTACATTCTCCTTTCTGATTGCATCTTTAACTGAAGGAACCACCCAGTTTTTTTTGTTATCAGGCGGATTAAACCCTATGCGCTGTATATAACTGGTGTTATTAAAATCTGATTCAGGTTTAGTTCCTGTTTCTGGATTGCCGGAATAATAGCAACTCAGGCCACGTAAAATATCGCCACCACGTTTATAACAATCCACCAAAATTTGCTCTGAAACCTTGAGATTTTCGCAGGGGTCAAACATTTTCTCAGCGGTTGTATTGAAGTTTGCAAAATTCGTACTCGTTATTTGCATAAGACCGACTGAGTAACGATGATTTCGTGATTCAATTTGATTTACGATCTGGAGTGCAGCCTCCTTAGTTTTGGGAAAGTAGGACACTACGCCTTTGTCACCGGGCTTACGTTCAACCTTCGGTATTATCTCGGCAATAGCATATGGGTTAAAACCAGATTCAACCCTTGCAACTTCATGCGCAGTATCGGGATGAACGCTGGCCGCGCATTGCAAAGCAAGCGCCGCAAAAGCAGTAGTAGATAACATGATTTTCTCCCGGCCAGAACAGCGCGGAAAGTTTAGACTGAATGGTTAGTCATCGAAGTCGTCATCGTCTTCATCGTCTTCGTTTATCAGCTCCTCAATAGCCTGGCTGGAATATTTTTTTATGAGGTACTGGACAACACCAGAGCTTGAAATACGTCGCCCCCTGCGATAGCCAATCTCCAGAGCAATTTTATCCAGTTCCTCACGAGAAGCTGAGGGCAGGTAGAGTGTATTACGTTGGGGCTTGCGTCTCGTTGCGGTTTTTTTTCGGGTAGCAGTCATTTTTCTTCCGTTTCATTTTGTGGGTTAAGCTTCTCTCTGTGATGTAGTGGATGAAAGCCATACTTCTTTGCTGCGGATTATCCATGAGTAGAATTTCAGCAAAATCGTGTGGAATTGATTTCCTTAAAAGATTTGCCTGAGCTGTTGTATGTGATTGTCTGTACAAAAGCTCCGCCATCACATCTTCTGGTACAGGTAAAGGCTCACCGCCAAATGAGATAAAGCGTTGTACGTAAGGGATAGCAGTTATTGTAGTGGAATGAACCTCTTCAAAATCTGCCTGGATAAAAAGATAACCGGGAAAAAGCGGCAAACGTCTTTCTCGATATGAAATTATCTTATCCGGGCGACGATACCTTTCAGTCTCCATAGGACAGAAGTAAGAGAAGTTCAGGCGTTCGAGATGAGCAAAAACTGCTGCAAATCTCGTAGTGGTGAATTGCAAAACATACCATTCCATAGTTTTTCCTTTCAGGCGCTCTCTCGACTCGGATGACCCCGAATCAGAGAAAAAAAACAAAAGGACAAATTTACATCTCATAAAAACTTTATCAGCTACATGTGCTGCGTTCAACCACACAAGCAGATCTTTAGATCGCATTTACGACACATTTCATTTTGTTTGTGCTTGTATTTTATTTATATCTAGATACATATGCTATTTGATAACAAAAAAGCAGATCGATTGATCACCATGCGCTTATTTTATAGTCAAGTTAACTAAATTTTTTACCGCTAGTTACTTTTTACAAAAACTCTGCTAACAAGATAAATAAATCGATCTTAATCAATAAGTTACCGGAGAGTATGAATTATCCTAAACGAATTCCGATTTTCACTTTCTGTTCGCTTTGAATCATCGACTGTTTTATTTGCGAGAAATTTCTCATCTGGAGTTGCTGTTTCTTAATCATCTCCGCGCTGTTCAGCACCAGCTCTTTACTGGATGAAGAGATTCTGTCTATGCCCTTAACCCCCAGGTTCTCTAGCTGCCACTCATTCCTGTGTGCAGTCTTCCAGCCATGCTGTACACCGTTCTTATCAAGTGCAGGGATTTTCACCTCCTTTTGTCCTAACTTCTTCAGCTTAACAAGATCACCTTTCTTAACGTTCTCGCGAGTGGTTAACTCACCAAAATCAGCCCCCCAATATGTTTTCTCCACGCCCTTGTTCAGTGTCTTAAGCTTTATGAAATGCTGCTTTGGTTTGGTTTTATCGTTCTGATAATGGTCGTAGCCAACATCAACAACCTCATAAACGCCCTTCTGTCGTTTTGGCGCTGTTTTGTGCGCATCTTTAATAGACTGGTTAAGTCCATGCTGCTGCTTATGAGTGGCTTTAACGTCATAGCCCTTCATCTTCAGCTCTTCACAAAAACCTGTACGAATTTCCCGTAAATCTTTTTTCCTGATATCAGCTCGTTTACCGTCATTATCACGAATACGAAAAACGACATGCACGTGTGGATGTTCTTTTTTGTCGTTATGATAGCCAAGCACAAAACGATGATTTGGGTACTTCTTGTTCATCGTTTTCCTGACAGACTCAAGCAGATCTTCAGGTTTTACCTTTGCTGATACAGGCGGTGAAAACACAATATTTTGGGTTACTTTTTTATTTTCCTTACCTTTATCATCGAAAACATTTTGAGGGTCATTAGCACGATCTATCATGTGCTCTTTAGCCTCCTGAATCTCATCACCTTTCCATACCTGACCGCTTTCACTCATCACTGGCAGCTCTGACTCCCGGCTCATATAGTCAATACTGTTTCGAACTCCCTGCCGGGTAATCGCACCTCCTGAGATTTTGACAACAACCTCCTTACTGGCACCCTTGCGGTTGATACGTTCCTGAACGTTGCGTTGATAGTTTTTACCACCATTTTTAACTTTATGGGCGAAAGCAGACTTGCGGCCAGCTTCAGAGGACTTACGCTTAACCCTGAATTCCTTGTCAACATAAACGCCCATGATTACACCTCAACCTTGTTAGAAACAGTTCGCCCTTTGCATAACATAATGTAGTTTTCAAACTCACTTTTGAGACTCATTATCTCTGAACTCAACCGCTCAGCGTCCTGATAGAACCCATCTTTATCAAGTACTCTGGCATTATCATTAACGATAATATAATGAAAGTTTCGCCCTATTTTATCTATTGCATTACGGCAACGATTCATCACCAGAAGTTCCTGATCGAAAAAATCAAGTTCATTTTCAAGTGTCGTCTGGATTCGATAACGACATTCTCTGGATAAATTCCAGCCATGACGATGAGCCGCTTTCTCTATTAAAGTTCTTTGTTTCCCTGTCAAATACATTGTGGTTTTGATAAAATCATCATTGTCTTTTGACACATTAACATCTTCATTTTTAACCTTGCTTTTCAGTAAAGCATTGGCAAGCAGCTTCATTCCTTTTGCCACGGTTAAACCCGGTTCATCTGGATAAAGTGTTTTAAGACAATCAAGTAGATCATCATGTTGGCTTTCTTCAATCCTGAATTGAACTTTTTTCATAAAACTCTCCAACAAGAACCGACTGTAGGTCACCGGGCAAACGTTGCGGAATGGCGTCAGAGACGTCATTTTGCGGCGTTTGCCCTATCCTGCATCGCAGTGAACTACCTCCACACATAAATTGTGCTTCAGCGTCCATCGAAATGCAAAAAATGAAAGGCTATCGTGGCTGTGATCTGGTGCGTCTGCGACCGGAGACAACGGATTCGCGGCTAACTTGGGCGGAAATAAATTCGCTACGCGAATGGCCTGGCAGGGGGCGCGAGCGCTGTTTTACGGAATATACAAAAAAAGCACCTCCCGTAAACGGGAGGGCTTCGGCGATTCAGGAACGGGAATTTTATTCTGCCTCTGGTGTGTCGCCTTCCATGCCCTGCCGGGCATCATGGAGCCAGCGGCGGAGGTTACTCATTCTTTCCCGGTTTGCCCGGCTGGGACTTTTTCCCCTGTTACCGCTTAGTATACTCCAGACTCTTAACCCGGCCTCGTTAGTCCAGTGCAATACAATTTCCCCTTTGCTGTTAAATCCCAGCGAAAACCGGACAGCTCCCCGCCTCTGTTCTGCACTGATAAAATCTAAAAACTCCTGTTTTTTCATAGCACCTCCTGTTTTTCTATCAGTGCTTTTGTTTCTTTCTTTTCCAGGGCTTTTCTGAGTAATGCCATGTACTCAGGAAAAGCAGAGAATGGCATCAAGGGAAAAACCGGACGCCCACCCTTTTCAACAGTCTGTATTGCTGATAACATGCGTTCCGACATTTTATATTCCTCGCGAGGGAATTGAGTGTCACGGTATCAGGTTGCCTCCACAACCTGATACCACCCCTAATTGATTAACTCCGCAGTTCTCTTATCGCTTCATTTTTGGCTTCCTCCAGTTCTTTAAGGGTATATGCGAAATTCTTCCAGTTAATTACTTCGTTTATAACCTCAGATTTACTTAAACCACACTCATTAAACCAAAAGTTAATATTTTTCTTGAGGGAATTTTTCGCCGTCAGTTCCTCGGACAACAATTCACTAACAGACGTGATAACATAACCACGACCGCGATTTTTATCTTCGAAAGAATGAACGGCAAGGAAGAGAGTTTCTGAATGGACAAAAGCAGAGCAGGTGATTTTTTCTTTCTTAGTGTTTAAAGCCTGGAAAGTGATAACATACGTGTTCATTTTAGTCTCCCGCCCGTTTGGGCTACTGGATCATGGTAAGGAACTATTCCCGACCTACAAATAAATTATACCATCATATGATGGTAATGCAATTTATTTAGTCACTTTATCCTGATTTTTTTCTCTCTTAACGAGCTGGTAATCTGGATGTTCATCAGCCAGGAGCAAAAGTAATTCATACGTACCAGCGGCTAAACCACGAGACGTTTTACCGGATGTTTCTTTGGCTTGCCAGGCTGATAACGTAAAACCAAACCGTTCAGCGGCTTCCTGCTGCGTAAAGCCAGCTTTAATACGTGCTTGTTTCACCTCTTCAGGTGTTGGCTTAAAACTCATGATTTTGTACCTTCACAATAGTATCTGTATAGAATTTCAATAAAATTGGATCATCAATTTCATCTTTAACGTTCGAGAATTCTTCGTAATAAAATGATTTTTGGAAAACAATCTTTTTAGTTGTTTTTTTATCCGCTTCTTTAGAGCGTACCTTCATCAGTTTTTCAAGTTCAATAAACAAACGTGTTGCATCTTCAGACACAACAATCCTGTATGTTACATTACCTTTGTTTTTCCTGTCGCTCAGGACTTCATTCATTTTTTGCCTCATAGCTGTTTAATAAACCCCGCTACCTTTATGAGCTGATCTACACTCAGGCCACGAATTACCATTTCATATCCGTCGCCCCGTTCTGATACGTTACCATCTAACCCATTCTCAACTTTAAACCCGGTCAATGTCCCATGCCAGCTTTCAAGATGCACTAAACGAGATAGCATTCCTTTCAGGATCAGTTTTTTCTCGCGTTTTTGCTGTAACTGTTGTTCATACTCTCGCGCCATATCTACATTATCTAAAGCATCCGACAGGATTTTCTTTTCAATATCGGCGGCGATTTCTACAGGGTTACGCTCTGGCGATACTGTGCAAGTATGGTAAGGACTTCTCCAGCTGCGACTATCTACGCTTCCAATGATAACTAACCTTCCTTTCTCCATTCGAATATGTATAGAGTAATTTTTAAAATCAGGTGAGGTTAGCTTAATCCTGTACTGGCAATCATCGAGCAGATTTACACGCCAGCCGTTCCCCAGGATTCGACAAACGATTTCAAACACCGGTTTATACCGTTCAAAGAAATTTTTCTGTTCTTTCATCGATTCAGCCTTTTGAACTCAATAAAAAAGCCGTAAATCGAATTACGGCAGTTTTAAGACTATTCATCAAAGTGTCCATCATCACTTGCAGCTTCCTGGGCTTCGCATTCCTGCTGATATGCTTCTTCCTTCGCAAAAAGATGCGCCCATCCTTCTGCGTTTAAGCCACGGTGCAAGACTGAACCATTTTCATTGCATTCTTTGGCTGTAACTTCGATCTCATCCGGGCAAATGTAGGCTCCGTAACTTTCAAAAATGTTTATCAGCTCCGCTTCCGTTATGGTCTTGTTTTTCATCAACGTGTCTCCGAATCAATCTTTGCCATGTTCTAGGGGGCTTTCGCCCCCATTCCGGTTATGCGCTTGCTTCTACACGTGTCCAGCCTGACGCCTTACGTTTACCCGCTGTACCATACATTTCACGAATTTCTTCAATGCTGTGTTCTTTACGGTTCCAGCGGCTTTTGTGTTCTTCAGGACGGTAAAACCATTGTTTTTTCTTAGATGCCCACTTACAGCCCATTTCCTTTAAAATGTCTTTGTGTTCTTTAGTTTCTCCGCTAATCCAGACCCAATTACCAATAACTTCATAGACAATCCCTGTAAGGCCGGAAAGCGTATTCAGAACTTTTTCCAGATCTTCACCGTAGTTGTAACGTGCGTTTTCATCAGTGCTTTGAAACTGATTGATTTTATCAATGTTAGCCATGAGAAAATCAAACGCAGCATTTACTGCTTTCATCAGCTCGGCACCCAATGGATTGCGGTCTGGATGATATTTTAAAGCCGCTTTTTTGTATGCCGCCTTGATGTCCTTTTCAGTCAGATCACCGGATAAACCAAAAACGTTTAATGCTTCCTGAATATTCATTTTTCGCTCCCGCCCGTGTGGGCTTCTGGAATCTGGCAGGGAACCATTCCCAACCAACAAATTCATTATACCATCAAATGATGGTTATGGAATCATTTTTAATGCTTTACATTGTAAAGATTACATAAAGATGTATATTGCTTTACCGCGCATGAAGGGATTTCCGAAAGAGGCGCGAACTGTAGTTACATAATGCGCTGTCGTAACATCGCGCCGGAGACGTTCGCCAGGACTGGCGAACCAACAAGCCCGAATGAGAGCGATAACCCCCCTCACCCCTGCCAGGCCAATTTCTGACTATCAGACGATGTTTAGCCTGTCGCAGTTAGCGCGTGAGCTTGCGAACGGTACGCCGGGTCAGTGTGAAACACTGGCGCGGGGCGCTGTATCTGCGCGTTATGAGATGCGTTAGCAGCGAATTACGGGGCGGGTTGCTGAAACCGATTGATGCCTGACAGCAAAAAATGAATCCGGGCGCCAGCTGCTGCACGAAAATTGCCAGCAAAAAACACTTGGCCATCCTCGCCGGTCGTGAAATTTTGCTGGCGTAAGAAATAATCCCCCCGGTATGAGGGGCTTGTCTGAAAGGTTAGTGGTTGGGCTTACTGAGAGTGAACTGTAACCCGGCTACTGCTGCAAGCTGTGCAAGTGTTGTGATGGTCGGGTTTCCGTTCGGGGATAATGCGCGGTAGATACTCTGACGAGCAAGGCCGGATTTTTTGGCCGCCTCTGTTATACCCCCTGATTAGGGATCAGGTTAAAAACGAGCTGTATGACGTTCTAAGCGCATTTTACCCGCTCCGTTGAACCATGACCGGGTTAAAGCGGTTAAAACGCCTCTGAGGGCTTTTGAGAGCATTTAAGAATTGGATCGGAAAAGGATCCCGTCTGGATCTTTTAGAGAGATTACACCGCTCGCCGCAGCCGAACGACAGGGCGAAGCCCGCGAGTGAGCGAGGAAGCGGAATTTCCACGGGGTTTCAATAGAGCACTTACGCGCTAAGGTTAATATTTGCTCAATTTTTGGTGGTGCAGTGCTCAGGGAGGCCAGACGGATCTATCCTCTGGATTTATGCTGAGTTATCCACAGACGCTGTGCATTAAAAGCCTTATATTTTCTTCTTGATCTTAAAAACTTAAAAACTTAGCTCGCTTCAATTACCTGAAAATTATGGAAAAAAATTGCTAAAGGTGAGAGGATTTTTCACGTAAGGTGAGAGGATTTTTCACGTAAGGTGAGAAGATTTTTAAGTTAAGGTAGTTAGATTTTTCACTAAAGATGAGAGGATTTTTCACGTAAGGTGAGAAGATTTTTAACGGTTTCCACAGTTTGACTGAACATCAAGTGAAAC
