# Supplementary material for: Novel Variant of New Delhi Metallo-β-lactamase, NDM-20, in Escherichia coli
Source: Front Microbiol. 2018 Feb 21;9:248. doi: 10.3389/fmicb.2018.00248 (PMC5826333; doi:10.3389/fmicb.2018.00248)
Supplement: TABLE S1 — Amplified and cloning primers. [file Table_1.DOCX]

**Table S1. Amplified and cloning primers**

| primers | sequences*^a^* |
| --- | --- |
| preA | 5’-CACCTCATGTTTGAATTCGCC-3’ |
| preB | 5’-CTCTGTCACATCGAAATCGC-3’ |
| EcoRI-NDM-F | 5’-CGGAATTCATGGAATTGCCCAATATTATG-3 |
| PstI-NDM-R | 5’-AACTGCAGTCAGTGCAGCTTGTCGGCCAT-3’ |
| BamHI-TEV-NDM-F | 5’-CGGGATCC**GAAAACCTGTATTTCCAAGGC**CAGCAAATGGAAACTGGCGAC-3’ |
| XhoI-NDM-R | 5’-CCGCTCGAGTCAGTGCAGCTTGTCGGCCATG-3’ |

*^a^*The sequences of restriction enzyme site were shown with underline and TEV protease recognition sequence was bold.
